# CRIP1 cooperates with BRCA2 to drive the nuclear enrichment of RAD51 and to facilitate homologous repair upon DNA damage induced by chemotherapy

**DOI:** 10.1038/s41388-021-01932-0

**Published:** 2021-07-14

**Authors:** Huiying Sun, Rui Zhou, Yannan Zheng, Zhaowei Wen, Dingling Zhang, Dongqiang Zeng, Jianhua Wu, Zhenhua Huang, Xiaoxiang Rong, Na Huang, Li Sun, Jianping Bin, Yulin Liao, Min Shi, Wangjun Liao

**Affiliations:** 1 Department of Oncology, Nanfang Hospital, Southern Medical University, Guangzhou, Guangdong PR China; 2 Department of Cardiology, Nanfang Hospital, Southern Medical University, Guangzhou, Guangdong PR China

**Keywords:** Cancer therapeutic resistance, Homologous recombination

## Abstract

Homologous recombination (HR) repair is an important determinant of chemosensitivity. However, the mechanisms underlying HR regulation remain largely unknown. Cysteine-rich intestinal protein 1 (CRIP1) is a member of the LIM/double-zinc finger protein family and is overexpressed and associated with prognosis in several tumor types. However, to date, the functional role of CRIP1 in cancer biology is poorly understood. Here we found that CRIP1 downregulation causes HR repair deficiency with concomitant increase in cell sensitivity to cisplatin, epirubicin, and the poly ADP-ribose polymerase (PARP) inhibitor olaparib in gastric cancer cells. Mechanistically, upon DNA damage, CRIP1 is deubiquitinated and upregulated by activated AKT signaling. CRIP1, in turn, promotes nuclear enrichment of RAD51, which is a prerequisite step for HR commencement, by stabilizing BRCA2 to counteract FBXO5-targeted RAD51 degradation and by binding to the core domain of RAD51 (RAD51^184–257^) in coordination with BRCA2, to facilitate nuclear export signal masking interactions between BRCA2 and RAD51. Moreover, through mass spectrometry screening, we found that KPNA4 is at least one of the carriers controlling the nucleo-cytoplasmic distribution of the CRIP1–BRCA2–RAD51 complex in response to chemotherapy. Consistent with these findings, RAD51 inhibitors block the CRIP1-mediated HR process, thereby restoring chemotherapy sensitivity of gastric cancer cells with high CRIP1 expression. Analysis of patient specimens revealed an abnormally high level of CRIP1 expression in GC tissues compared to that in the adjacent normal mucosa and a significant negative association between CRIP1 expression and survival time in patient cohorts with different types of solid tumors undergoing genotoxic treatments. In conclusion, our study suggests an essential function of CRIP1 in promoting HR repair and facilitating gastric cancer cell adaptation to genotoxic therapy.

## Introduction

Although chemotherapy achieved a significant improvement in overall survival (OS) vs. surgery alone for patients with locally advanced resectable gastric cancer (GC), the 5-year survival rate remains limited (only 36% in the MAGIC Trial) [1], indicating that additional efforts are required to enhance treatment effectiveness.

Inducing DNA double-strand breaks (DSBs), the most hazardous DNA lesions [2], is the main mechanism for chemotherapeutic agents, especially anthracycline and platinum, to exert cytotoxic effects. Non-homologous end-joining (NHEJ) and homologous recombination (HR) pathways are the major pathways for DSB repair [3, 4]. Unlike NHEJ, which is an error-prone process leading to chromosomal translocation and genome instability [5], HR is an error-free repair mechanism for eliminating DSBs, in which the homologous sequence of an intact sister chromatid is used as a template for repair synthesis [4]. Therefore, although the HR only deals with a minority of DSBs, it is the most crucial DSB repair pathway because of its high fidelity. Growing evidence has revealed the close connection between chemotherapy sensitivity and HR deficiency [6]. However, to date, the precise regulatory mechanisms of HR pathways still remain largely unknown.

Cysteine-rich intestinal protein 1 (CRIP1), a member of the LIM/double-zinc finger protein family, is overexpressed and associated with prognosis in several tumor types [7–11]. However, its functional role functional role of CRIP1 in cancer biology is poorly understood. Recent studies have revealed that CRIP1 may have tumor type-specific oncogenic or tumor suppressive properties [10–12]. Regarding GC, only one article has reported that high CRIP1 expression is an independent predictor of shortened survival in patients with intestinal disease [7]. Herein, we clarified an unreported mechanism wherein CRIP1 enhances the HR repair pathway by activating the BRCA2–RAD51 axis to facilitate tumor cell adaptation to lethal DNA breaks.

## Results

### CRIP1 overexpression is linked to worse prognosis in patients undergoing genotoxic treatment

**Fig. 1:**
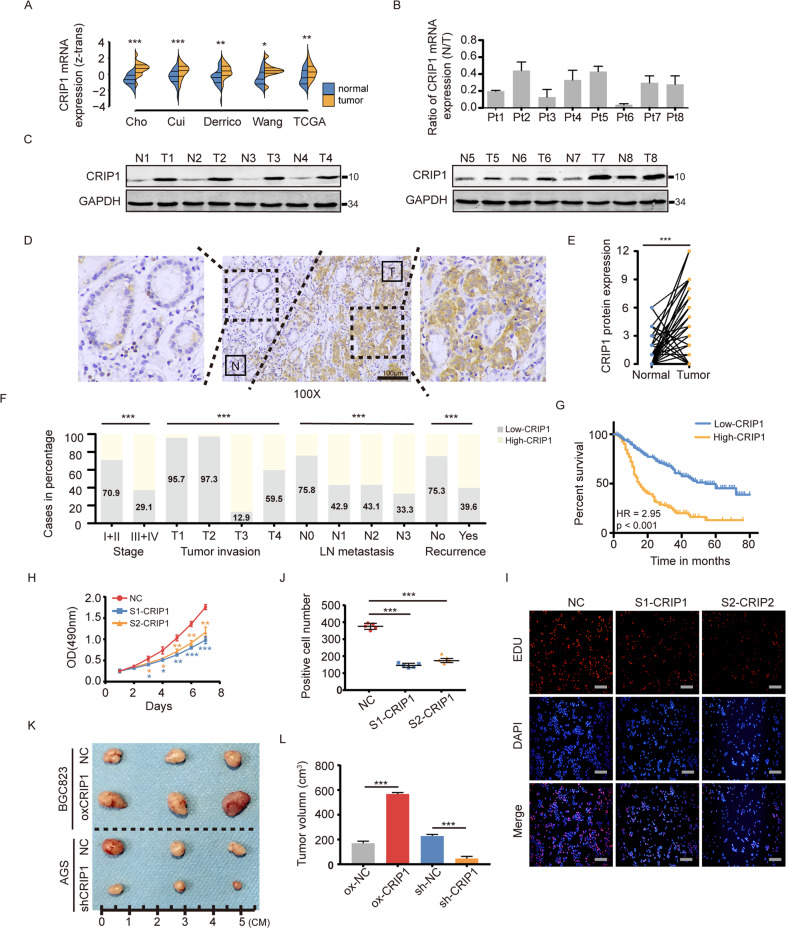
CRIP1 is overexpressed in gastric cancer (GC). **A** Analysis of CRIP1 mRNA expression data from Oncomine GC database (*p*-values from left to right: *p* = 9.00e − 6, *p* = 3.3e − 5, *p* = 0.0013, *p* = 0.027, and *p* = 0.0042). **B**, **C** CRIP1 expression evaluated by real-time PCR (**B**) and western blotting (**C**) in eight pairs of GC tissues and adjacent noncancerous tissues. **D** Representative micrographs of CRIP1 protein expression in GC and normal gastric tissues, as detected by immunohistochemistry. Scale, 100 μm. **E** Quantification of immunohistochemical staining intensity in normal gastric tissue and paired GC tissues (*p* = 1.90e − 38). **F** Bar charts summarizing proportions of patients with low-CRIP1 expression within and across groups categorized by TNM stage, tumor invasion, lymph node metastasis, and recurrence status. The *χ*^2^-tests were used for statistical comparisons (*p*-values from left to right: *p* = 1.14e − 8, *p* = 4.24e − 22, *p* = 9.77e − 7, *p* = 2.40e − 8). **G** Kaplan–Meier curves of disease-free survival according to CRIP1 protein expression groups (stained by IHC assay) in the Nanfang hospital cohort. **H** Proliferation of GC cells transfected with empty vector or CRIP1 siRNA, as determined by MTT assay. The mean ± SD of five replicates of each time point were shown. **I**, **J** Proliferation of AGS cells transfected with empty vector or CRIP1 siRNA, as determined by EdU assay (**I**) and quantification of the number of positive cell number (**J**). The mean ± SD of five replicates were shown (*p*-values from left to right: *p* = 2.9e − 5, *p* = 2.0e − 5). Scale, 100 μm. **K**, **L** Representative images (**K**) and quantification (**L**) of transplanted subcutaneous tumors from mouse models. The mean ± SD of three replicates were shown (*p*-values from left to right: *p* = 5.0e − 6, *p* = 1.4e − 4). CT, chemotherapy; N, normal; T, tumor; LN, lymph node; NC, negative control; HR, hazard ratio; oxCRIP1, CRIP1 overexpression; *p < 0.05; **p < 0.01; ***p < 0.001.

We first analyzed public gene expression datasets and patient specimens from our hospital, and found that CRIP1 mRNA and protein expressions were both significantly higher in tumors than in noncancerous gastric tissue (Fig. 1A–C). Immunohistochemistry (IHC) assays (Fig. 1D, E and Supplementary Fig. S1A, B) further showed that CRIP1 was overexpressed in both the cytoplasm and the nucleus of GC cells, as well as in the tumor mesenchyme. Clinical data analysis showed that CRIP1 expression was higher in cases of more advanced disease (Fig. 1F), whereas there was no difference in CRIP1 expression between intestinal and diffuse type disease (Supplementary Fig. S1C). Survival analyses revealed a negative association between CRIP1 expression and survival time in GC patient cohorts administered adjuvant chemotherapy (Fig. 1G, Table 1, and Supplementary Fig. S1D). Interesting, analysis of the Kaplan–Meier plotter online database revealed a correlation between poor OS and elevated CRIP1 gene expression could be observed in cohorts with gastric, lung, breast, or ovarian cancer patients administered chemotherapy or radiotherapy (Supplementary Fig. S1E), indicating that CRIP1 overexpression might be used as a universal indicator of poor prognosis in multiple cancers when patients are treated with cytotoxic therapy.

**Table 1:** Univariate and multivariate survival analyses of CRIP1 and clinical variables.

	UVA		MVA	
	HR (95% CI)	*p*-Value	HR (95% CI)	*p*-Value
Age^a^	0.92 (0.67–1.26)	0.603	0.99 (0.71–1.38)	0.947
Gender (vs. male)	0.98 (0.71–1.36)	0.918	1.00 (0.71–1.41)	0.994
CRIP1 group (vs. lower group)	2.95 (2.15–4.07)	<0.001	2.38 (1.69–3.37)	<0.001
Stage (vs. stage II)	2.46 (1.78–3.39)	<0.001	1.89 (1.33–2.70)	<0.001
Grade (vs. poor)	0.86 (0.67–1.11)	0.254	0.97 (0.74–1.36)	0.795

*CI* confidence interval, *HR* hazard ratio, *MVA* multivariate analysis, *UVA* univariate analysis.

^a^Continuous variable.

To further confirm the oncogenic properties of CRIP1 in GC, we silenced the CRIP1 expression in AGS and BGC823 cell lines using siCRIP1 (Supplementary Fig. S1F–I) and found that the downregulation of CRIP1 led to a significant inhibition of cell proliferation (Fig. 1H–J and Supplementary Fig. S1J–L). We then constructed CRIP1-stable silencing cells (AGS cells transfected with shCRIP1) and overexpressing (BGC823 cells transfected with lentivirus) cell lines to investigate the effect of CRIP1 on tumor progression in vivo (Supplementary Fig. S1M, N). Subcutaneous tumor growth was clearly decreased by silencing CRIP1 and increased with CRIP1 overexpression in tumor cells, further supporting the argument that CRIP1 acts as an oncogene in GC (Fig. 1K, L).

### Downregulation of CRIP1 results in attenuation of DDR and increase in chemotherapy sensitivity in GC cells

**Fig. 2:**
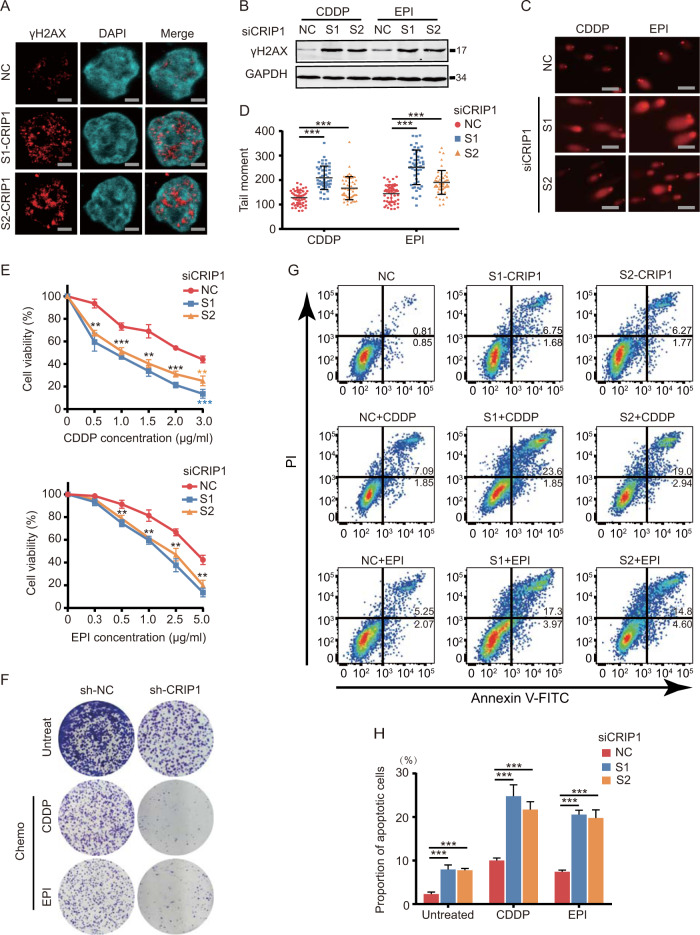
CRIP1 knock down inhibits DNA repair and increases susceptibility to chemotherapy in AGS cells. **A** Representative images of γH2AX staining of AGS cells transfected with empty vector or CRIP1 siRNA after cisplatin withdrawal. Scale, 2.5 μm. **B** Western blot analysis of γH2AX protein level in AGS cells transfected with empty vector or CRIP1 siRNA after cisplatin (1 μg/ml, treated for 24 h before withdrawal) or epirubicin (1 μg/ml, treated for 24 h before withdrawal) withdrawal. **C**, **D** Neutral comet assay measure of DNA damage in AGS cells transfected with empty vector or CRIP1 siRNA under stimulation of cisplatin or epirubicin. Representative image (**C**) and quantification of tail moments (**D**) were shown. Fifty replicates were used (the *p*-values from left to right: *p* = 4.10e − 17, *p* = 3.0e − 6, *p* = 1.13e − 25, *p* = 6.20e − 7). Scale, 100 μm. **E** Dose–response curves of AGS cells transfected with empty vector or CRIP1 siRNA after treatment with cisplatin or epirubicin for 24 h. The mean ± SD of five replicates of each time point were shown. **F** Colony formation ability of shCRIP1 and corresponding control AGS cells with or without chemotherapeutic drug treatment. **G** Flow cytometry analysis of apoptosis of AGS cells transfected with empty vector or CRIP1 siRNA under stimulation of vehicle or chemotherapeutic drugs. The sum of Q2 and Q3 represents the total percentage of early and late apoptotic cells. **H** The proportions of apoptotic cells were displayed by the bar chart. The mean ± SD of three replicates of each time point were shown. CDDP, cisplatin; EPI, epirubicin; NC, negative control. *p < 0.05; **p < 0.01; ***p < 0.001.

CRIP1 was reportedly upregulated by ultraviolet radiation in primary human keratinocytes [13]. As ultraviolet light induces for DNA damage, we predicted that CRIP1 mediates DNA damage repair (DDR) processes. As expected, the remaining γH2AX foci, the γH2AX expression levels, and the comet tail lengths were all increased in AGS (Fig. 2A–D and Supplementary Fig. S2A) and BGC823 cells (Supplementary Fig. S2A–E) following CRIP1 silencing on the fifth day after chemotherapeutic drug withdrawal (cisplatin (CDDP): 1 μg/mL, epirubicin (EPI): 1 μg/mL, treated for 24 h before withdrawal), indicating that cells lacking CRIP1 harbor defects in DNA repair. As DNA repair efficiency is associated with chemoresistance, we next investigated whether CRIP1 affects the chemosensitivity of EPI and CDDP, representative drugs that induce DSBs. The results of MTT (3-(4, 5-dimethylthiazolyl-2)-2, 5-diphenyltetrazolium bromide) (Fig. 2E and Supplementary Fig. S2F), cell colony formation (Fig. 2F and Supplementary Fig. S2G), and flow cytometry assays (Fig. 2G, H and Supplementary Fig. S2H–I) all showed that EPI and CDDP both exhibited stronger cytotoxicity in GC cells with CRIP1 silencing, suggesting that CRIP1 is important for maintaining genome stability and is required for cell survival following DNA damage.

### CRIP1 modulates HR repair activity and GC cell sensitivity to the PARP inhibitor

**Fig. 3:**
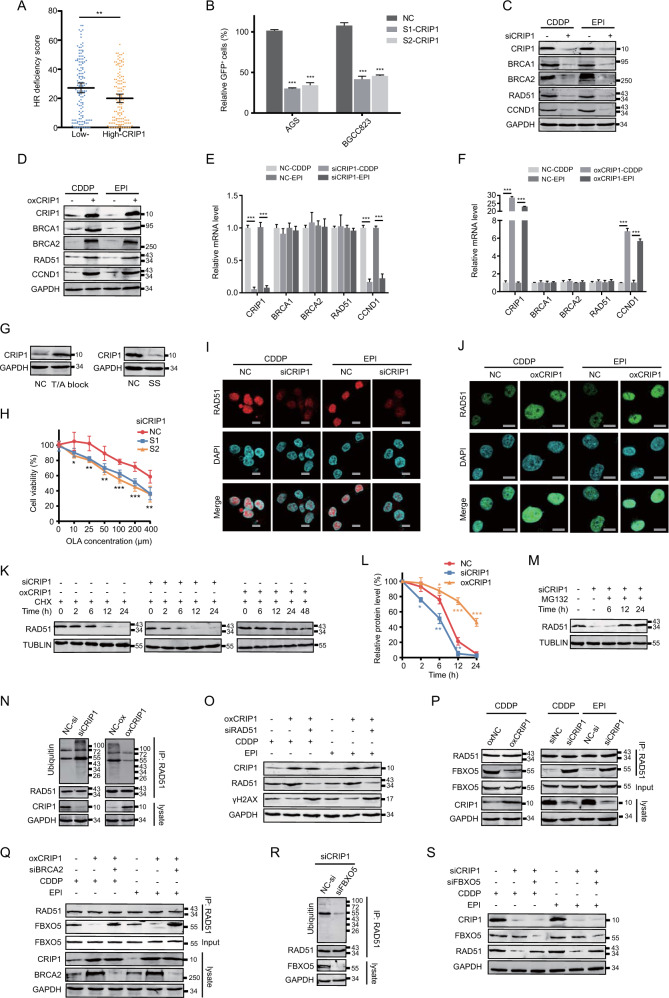
CRIP1 is required for homologous recombination (HR) repair in AGS cells. **A** The distribution of HR deficiency score in gastric cancer patient groups with high- and low-CRIP1 expression in the TCGA dataset (*p* = 0.002). **B** HR repair assays of AGS and BGC823 DR-GFP cells transfected with empty vector or CRIP1 siRNA. AGS and BGC823 cells stably expressing the DR-GFP plasmid were transfected with empty vector or CRIP1 siRNA for 24 h. The cells were then co-transfected with I-SceI. At 48 h post transfection, the percentage of GFP-positive cells was measured by flow cytometry. The mean ± SD of three independent experiments were shown (the *p*-values from left to right: *p* = 5.84e − 9, *p* = 9.01e − 7, *p* = 1.3e − 5, *p* = 3.0e − 6). **C**, **D** Western blot analysis of the protein levels of molecules involved in BRCA2–RAD51 axis in CRIP1-knockdown (**C**) or -overexpressing (**D**) AGS cells as compared to the control group after cisplatin or epirubicin treatment. **E**, **F** Real-time PCR analysis of the mRNA level of molecules involved in BRCA2–RAD51 axis in CRIP1-knockdown (**E**) or -overexpressing (**F**) AGS cells as compared to the control group after cisplatin or epirubicin treatment. The mean ± SD of three replicates were shown. **G** Western blot analysis of CRIP1 expression in AGS cells treated with thymidine/aphidicolin block (left) or serum starvation (right). **H** Dose–response curves of AGS cells transfected with empty vector or CRIP1 siRNA after treatment with olaparib in different concentration for 24 h. The mean ± SD of five replicates of each time point were shown. **I**–**J** Immunofluorescence assays of RAD51 expression and foci formation in CRIP1-silencing (**I**) or -overexpressing (**J**) AGS cells as compared to the control group after cisplatin or epirubicin treatment. Scale bar, 10 μm. **K**–**L** Western blot analysis of RAD51 protein level (**K**) and quantification of blot intensity (**L**) in AGS cells transfected with empty vector, CRIP1 siRNA, or CRIP1 plasmid at different time points after cycloheximide treatment. The western blot assays for the protein samples in the siCRIP1, oxCRIP1, and empty vector groups were performed separately in different gels. The loading amount and exposure intensity for the protein sample of each group were adjusted to ensure clarity of the blots. At 48 h post transfection, cycloheximide was added into cell culture medium to block endogenous protein synthesis. The relative protein expression level of RAD51 protein was determined as the relative blot intensity of RAD51 to that of TUBLIN at each time point and was set as 1 for cells at the time 0 h. The mean ± SD of three replicates of each time point were shown. **M** Western blot analysis of RAD51 protein level in CRIP1-knockdown AGS cells treated with vehicle or MG132. The whole-cell lysate of AGS cells transfected with empty vector was used as a control for the expression level of RAD51. **N** Co-immunoprecipitation analysis of RAD51 ubiquitination level in CRIP1-knockdown or -overexpressing AGS cells as compared to the control group. RAD51 was immunoprecipitated and blots of endogenous ubquitination were probed with the ubiquitin antibody. **O** Western blot analysis of CRIP1, RAD51, and γH2AX protein levels in AGS cells transfected with CRIP1 plasmid alone or co-transfected with CRIP1 plasmid and RAD51 siRNA under chemotherapeutic drug stimulation. **P** Co-immunoprecipitation analysis of the interaction between RAD51 and FBXO5 in CRIP1-knockdown or -overexpressing AGS cells as compared to the control group under chemotherapeutic drug stimulation. **Q** Co-immunoprecipitation analysis of the interaction between RAD51 and FBXO5 in AGS transfected with CRIP1 plasmid alone or co-transfected with CRIP1 plasmid and BRCA2 siRNA under chemotherapeutic drug stimulation. **R** Co-immunoprecipitation analysis of RAD51 ubiquitination level in AGS cells co-transfected with CRIP1 siRNA and FBXO5 siRNA as compared to the control group. RAD51 was immunoprecipitated and blots of endogenous ubquitination were probed with the ubiquitin antibody. **S** Western blot analysis of CRIP1, RAD51, and FBXO5 protein levels in chemotherapeutic drug-treated AGS cells, which were transfected with CRIP1 siRNA alone or co-transfected with CRIP1 siRNA and FBXO5 siRNA as compared to the control group. CDDP, cisplatin; EPI, epirubicin; NC, negative control; SS, serum starvation; T/A, thymidine/aphidicolin; oxCRIP1, CRIP1 overexpression. *p < 0.05; **p < 0.01; ***p < 0.001.

We determined whether CRIP1 participates in the HR process to render GC cells resistant to CDDP and EPI. By studying the The Cancer Genome Atlas (TCGA) GC cohort, we found that the HR deficiency score [14] was significantly decreased in tumor samples with high *CRIP1* expression (Fig. 3A), supporting the role of CRIP1 in maintaining HR function in GC cells. This finding was corroborated by the results of plasmid-based HR repair reporter assays, which revealed that the CRIP1 silencing in GC cells significantly decreased (>60% for AGS and BGC823) the percentage of green fluorescent protein (GFP)-positive cells upon I-SceI expression (Fig. 3B). In addition, the protein expression levels of the several HR effectors, including BRCA1, BRCA2, RAD51, and CCND1, were decreased following CRIP1 silencing, and were increased after CRIP1 exogenous overexpression (Fig. 3C, D and Supplementary Fig. S3A). However, at the mRNA level, no obvious difference was observed between groups with CRIP1 mRNA interference and the corresponding control, except in CCND1 expression (Fig. 3E, F Supplementary Fig. S3B, C). Interestingly, the total expression levels and phosphorylation levels of RPA2 and CtIP (both involved in DNA resection) remained largely unchanged after both siCRIP1 treatment and CRIP1 overexpression (Supplementary Fig. S3D). It is widely accepted that there is a relationship between HR repair and the cell cycle, wherein HR is mainly activated in the S phase, whereas NHEJ is activated mainly at the G0/G1 phase. Thus, we induced cell cycle synchronization at the S phase by a thymidine/aphidicolin (T/A) block or G1 phase by serum starvation, to observe CRIP1 expression in GC cells at different points in the cell cycle. CRIP1 expression was increased substantially by the T/A block, but was inhibited by serum starvation (Fig. 3G and Supplementary Fig. S3E). Finally, through MTT assays, we uncovered a negative correlation between the CRIP1 expression level and sensitivity to the poly ADP-ribose polymerase (PARP) inhibitor olaparib in GC cells (Fig. 3H).

### CRIP1 counteracts FBXO5-dependent RAD51 degradation to maintain adequate RAD51 protein levels

RAD51 is a central factor in HR repair and its nuclear enrichment is a prerequisite step for HR commencement [15]. Thus, we monitored RAD51 recruitment to the cell nucleus and DNA break sites, and found that both the RAD51 nuclear protein level and foci formation (a biomarker for HR function assessment) after drug treatment were obviously blunted by CRIP1 silencing and increased by CRIP1 overexpression (Fig. 3I, J and Supplementary Fig. S3F, G). As CRIP1 regulates RAD51 expression at the posttranscriptional level (Fig. 3C–F and Supplementary Fig. S3A–C), we used cycloheximide to inhibit protein synthesis and found that the protein degradation rate of RAD51 was dramatically reduced by introduction of CRIP1, but accelerated after siCRIP1 treatment, which could be neutralized by MG132 treatment (Fig. 3K–M and Supplementary Fig. S3H–J). Correspondingly, the ubiquitination of RAD51 was also dramatically elevated by CRIP1 deficiency and decreased by CRIP1 overexpression (Fig. 3N and Supplementary Fig. S3K). In addition, RAD51 silencing in CRIP1-overexpressing cells further validated that CRIP1 regulates HR repair in a RAD51-dependent manner (Fig. 3O and Supplementary Fig. S3L).

RAD51 is reportedly ubiquitinated and degraded in an FBXO5-dependent manner in cells lacking BRCA2 expression [16]. Given that silencing CRIP1 downregulates both BRCA2 and RAD51, we hypothesized that CRIP1 silencing may strengthen the RAD51–FBXO5 interaction to de-stabilize RAD51. As expected, although CRIP1 itself did not affect the total protein expression levels of FBXO5 (Supplementary Fig. S3D), binding of FBXO5 to RAD51 was clearly inhibited by CRIP1 overexpression and enhanced by CRIP1 silencing (Fig. 3P and Supplementary Fig. S3M). Moreover, co-silencing of BRCA2 restored the weakened RAD51–FBXO5 interaction induced by CRIP1 overexpression, suggesting that CRIP1 counteracts FBXO5-dependent RAD51 degradation by stabilizing BRCA2 (Fig. 3Q and Supplementary Fig. S3N). Finally, co-silencing of FBXO5 in CRIP1-knockdown cells attenuated the ubiquitination levels of RAD51 and rescued RAD51 protein levels (Fig. 3R, S and Supplementary Fig. S3O, P), suggesting that CRIP1 regulates RAD51 stability at least partly through the FBXO5-dependent degradation pathway.

### CRIP1 binds to the core domain of RAD51 in coordination with BRCA2 to facilitate NES-masking interactions between BRCA2 and RAD51

**Fig. 4:**
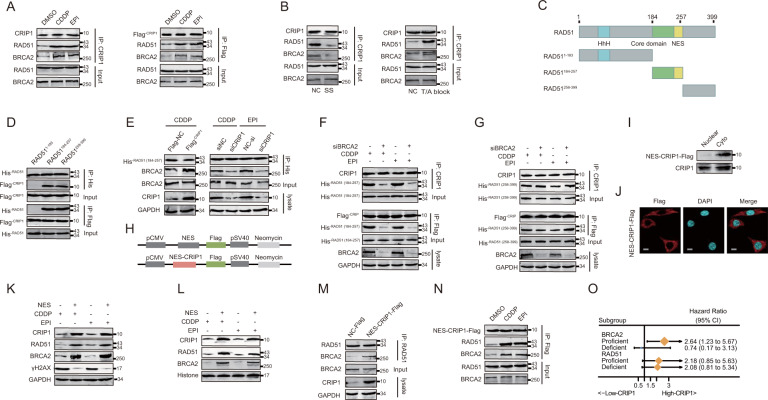
CRIP1 interacts with BRCA2–RAD51 complex in AGS cells. **A** Co-immunoprecipitation analysis of interactions of endogenous CRIP1 (left) and exogenous flag-tagged CRIP1 (right) with RAD51 and BRCA2 in AGS cells treated with vehicle or chemotherapeutic drugs. **B** Co-immunoprecipitation analysis of interactions of CRIP1 with RAD51 and BRCA2 in AGS cells treated with serum starvation (left) or thymidine/aphidicolin block (right). **C** Schematic of the his-tagged RAD51 fragments. **D** Co-immunoprecipitation analysis of interactions of flag-tagged CRIP1 with his-tagged RAD51 fragments in AGS cells. **E** Co-immunoprecipitation analysis of the interactions between RAD51^184–257^ and BRCA2 in AGS cells transfected with flag-tagged CRIP1 or CRIP1 siRNA as compared to the control group under chemotherapeutic drug stimulation. **F**, **G** Co-immunoprecipitation analysis of interactions of endogenous CRIP1 (upper) and exogenous flag-tagged CRIP1 (down) with RAD51^184–257^ (**F**) and RAD51^258–399^ (**G**) in AGS cells transfected with empty vector or BRCA2 siRNA under chemotherapeutic drug stimulation. **H** Flag-tagged NES sequence-fused vector and flag-tagged NES sequence-fused CRIP1 proteins were depicted graphically and color coded. **I**–**J** Western blot assay (**I**) and immunofluorescence assay (**J**) to detect subcellular localization of NES-CRIP1-Flag protein in AGS cells. Scale bar, 10 μm. **K**, **L** Western blot analysis of HR-related proteins in cytoplasm (**K**) and nucleus (**L**) of AGS cells transfected with empty vector or NES-CRIP1-Flag construction. **M** Co-immunoprecipitation analysis of the interaction between RAD51 and BRCA2 in AGS cells transfected with empty vector or NES-CRIP1-Flag. **N** Co-immunoprecipitation analysis of the interaction between NES-CRIP1-Flag and BRCA2–RAD51 complex in AGS cells treated with vehicle or chemotherapeutic drugs. **O** Forest plot of the associations between CRIP1 expression and overall survival in various subgroups stratified by BRCA2 and RAD51 expression levels. Unadjusted HRs (boxes) and 95% confidence intervals (horizontal lines) were depicted. DMSO, dimethyl sulphoxide; NES, nuclear export signal; CDDP, cisplatin; EPI, epirubicin; CI, confidence interval; NC, negative control; SS, serum starvation; T/A, thymidine/aphidicolin.

RAD51 forms a stable complex with BRCA2 and this interaction is essential for RAD51 nuclear localization [15, 17]. CRIP1 contains a cysteine-rich LIM domain, which plays an important role in mediating protein interactions [18, 19]. Therefore, we next examined whether there is also a physical interaction between CRIP1 and the BRCA2–RAD51 complex, and whether CRIP1 drives RAD51 nuclear accumulation by facilitating the BRCA2–RAD51 interaction. Co-immunoprecipitation assays revealed that both endogenous CRIP1 and exogenous flag-tagged CRIP1 interacted with RAD51 and BRCA2 (Fig. 4A and Supplementary Fig. S4A). These interactions were detected via protein extraction of both chemotherapeutic drug-treated and -untreated cells, and were enhanced by CDDP or EPI stimulation (Fig. 4A and Supplementary Fig. S4A), indicating that such interactions did not rely on but were modulated by DNA damage signals. Similarly, we also found that the interaction among CRIP1, BRCA2, and RAD51 was also increased by a T/A block, whereas serum starvation disrupted such interactions (Fig. 4B and Supplementary Fig. S4B). Moreover, when co-expressing flag-tagged CRIP1 with various RAD51 fragments (Fig. 4C) in GC cells, we noticed that the bindings of RAD51^184–257^ and RAD51^258–399^ to CRIP1 remained, whereas that of RAD51^1–183^ was impaired (Fig. 4D and Supplementary Fig. S4C). Among the above fragments, RAD51^184–257^ contains the core domain that has been implicated in BRCA2 binding [20] and controlling RAD51 nuclear accumulation. In detail, the nuclear export signal (NES) motif (spanning amino acids 245–260) lying within the RAD51 core domain becomes masked when the protein is bound to BRCA2 in the cytoplasm to permit nuclear localization [15]. As CRIP1 also bound to RAD51^184–257^, we next aimed to determine whether CRIP1 competes with BRCA2 for binding with RAD51^184–257^. We found that binding between RAD51^184–257^ and BRCA2 strengthened after flag-CRIP1 overexpression but decreased in siCRIP1-treated cells following DNA damage (Fig. 4E and Supplementary Fig. S4D). In addition, in BRCA2-knockdown GC cells, the CRIP1/RAD51^184–257^ interaction was also weakened, whereas the binding between CRIP1 and RAD51^258–399^ was not influenced (Fig. 4F, G and Supplementary Fig. S4E, F). These results indicate a specific synergistic relationship between BRCA2 and CRIP1 for binding to the core domain of RAD51.

To further ensure that CRIP1 participates in the BRCA2-mediated nuclear import of RAD51 in the cytoplasm, we generated an NES-CRIP1-Flag construct containing the NES motif (Fig. 4H), which generated a protein present exclusively within the cytoplasm of GC cells (Fig. 4I, J and Supplementary Fig. S4G). The cDNA sequence coding the NES peptide was obtained from Huang et al. [21] and was synthesized, annealed, and inserted into the flag-CRIP1 construct. The transfection of NES-CRIP1-Flag caused a dramatic upregulation in the total and nuclear expression of RAD51 and BRCA2, and enhancement of RAD51/BRCA2 interaction, but a significant decrease in γH2AX expression in GC cells treated with chemotherapeutic drugs (Fig. 4K, L and Supplementary Fig. S4H, I). Moreover, NES-CRIP1-Flag interacted with RAD51 and BRCA2 in AGS and BGC823 cells (Fig. 4M, N and Supplementary Fig. S4J, K). Interestingly, by analyzing a patient cohort being administered chemotherapy in the GSE62254 dataset, we noticed that the significant correlation between poor survival and elevated CRIP1 expression was affected by the transcriptional abundance of BRCA2 but not that of RAD51 (Fig. 4O), which presents further evidence that the impact of CRIP1 on the chemotherapeutic benefits of GC patients depends on a BRCA2-mediated HR repair process.

### KPNA4 assists the translocation of cytoplasmic CRIP1 into the nucleus along with the nuclear import of the BRCA2–RAD51 complex upon DNA damage

**Fig. 5:**
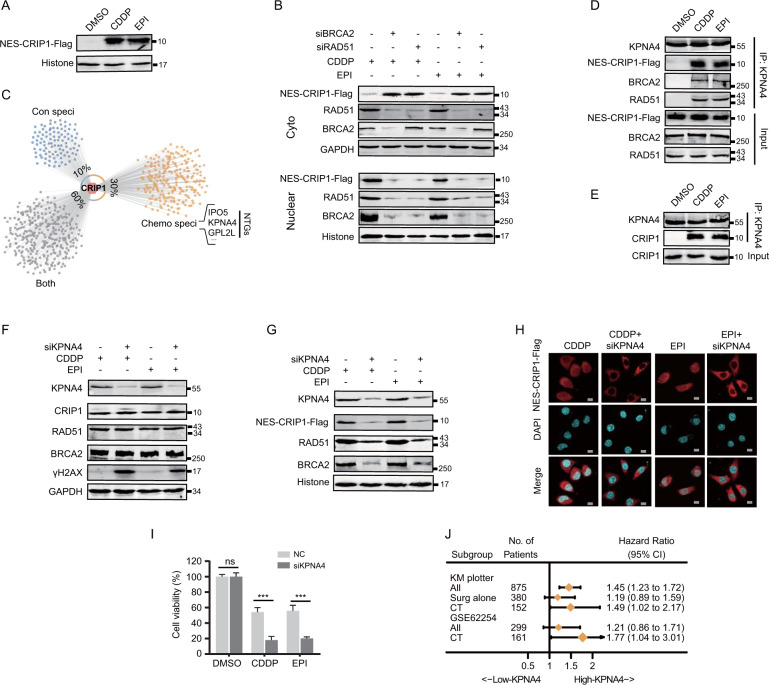
KPNA4 assists nuclear translocation of CRIP1/BRCA2/RAD51 complex during DNA damage response in AGS cells. **A** Western blot analysis of protein level of NES-CRIP1-Flag in the nucleus of AGS cells treated with vehicle or chemotherapeutic drugs. **B** Western blot analysis of NES-CRIP1-Flag, RAD51, and BRCA2 protein levels in the cytoplasm and nucleus in AGS cells transfected with BRCA2 siRNA or RAD51 siRNA as compared to the control group under chemotherapeutic drug stimulation. **C** Scatter plot shows the proteins interacted with endogenous CRIP1 as detected by mass spectrographic analysis. In summary, a total of 1000 proteins were identified as the potential interacting proteins of CRIP1. Among these proteins, 600 of them (30%, represented by orange dots) were only detected in the immunoprecipitate of cisplatin-treated AGS cells, whereas other 200 proteins (10%, represented by blue dots) were specifically detected in the immunoprecipitate of DMSO-treated AGS cells. The remaining 1200 proteins (60%, represented by gray dots) were detected in immunoprecipitates of both DMSO-treated and cisplatin-treated cells. **D** Co-immunoprecipitation analysis of the interactions between KPNA4, NES-CRIP1-Flag, and HR-related proteins in AGS cells treated with vehicle or chemotherapeutic drugs. **E** Co-immunoprecipitation analysis of the interaction between KPNA4 and endogenous CRIP1 in AGS cells treated with vehicle or chemotherapeutic drugs. **F**–**G** Western blot analysis of HR-related proteins in the cytoplasm (**F**) and nucleus (**G**) of AGS cells transfected with empty vector or KPNA4 siRNA under chemotherapeutic drug stimulation. **H** Immunofluorescence assay to detect subcellular localization of NES-CRIP1-Flag protein in AGS cells transfected with empty vector or KPNA4 siRNA under chemotherapeutic drug stimulation. Scale bar, 10 μm. **I** Cell viability of AGS cells transfected with empty vector or KPNA4 siRNA after treatment with vehicle or chemotherapeutic drugs for 24 h as determined by MTT assay. The mean ± SD of five independent experiments were shown (the *p*-values from left to right: *p* = 0.99, *p* = 5.0e − 6, *p* = 4.0e − 6). **J** Forest plots of the associations between KPNA4 expression and overall survival in various subgroups of “Kaplan–Meier plotter” online database and GSE62254 datasets. Unadjusted HRs (boxes) and 95% confidence intervals (horizontal lines) were depicted. Con, control; Chemo, chemotherapy; NTGs, nuclear transportation genes; NES, nuclear export signal; DMSO, dimethyl sulphoxide; CDDP, cisplatin; EPI, epirubicin; CT, chemotherapy; CI, confidence interval; NC, negative control. ***p < 0.001.

Nuclear transportation of proteins greater than 60 kDa requires the assistance of nuclear transport proteins [22]. Although CRIP1 (8.5 kDa) may pass freely into the nucleus, the protein complex formed by CRIP1, RAD51, and BRCA2 is unlikely to equilibrate passively across nuclear pores. Consistently, we discovered that the NES-Flag-CRIP1 was translocated to the nucleus only when the cells were treated with chemotherapeutic drugs and this nuclear import was blocked by silencing BRCA2 or RAD51 (Fig. 5A, B and Supplementary Fig. S5A, B), indicating that the nuclear translocation of CRIP1 is triggered passively along with the nuclear import of the BRCA2–RAD51 complex during genotoxic stress. To identify potential nuclear transporters controlling the nucleo-cytoplasmic distribution of the CRIP1–BRCA2–RAD51 complex, we performed a mass spectrometry (MS) analysis on the immunoprecipitates from CDDP- and DMSO-treated GC cells (Fig. 5C). Of all potential CRIP1 interactors we identified, 600 of them, which included several nuclear transporters, were only detected in the immunoprecipitates of CDDP-treated cells. Subsequent co-immunoprecipitation assays confirmed that KPNA4, a member of the importin family, could bind to the CRIP1–BRCA2–RAD51 complex only when cells were treated with EPI or CDDP (Fig. 5D, E and Supplementary Fig. S5C, D). Moreover, we found that KPNA4 silencing exerted no effect on total CRIP1, BRCA2, and RAD51 expression levels, but significantly decreased the nuclear accumulation of these proteins, delayed γH2AX clearance, and enhanced chemotherapy-induced apoptosis (Fig. 5F–I and Supplementary Fig. S5E–G). Consistent with the observed DDR promotion in vitro, GC patients with higher KPNA4 expression also presented shorter OS after receiving chemotherapy (Fig. 5J). These results suggested that KPNA4 is also indispensable for the functional integrity of cytoplasmic CRIP1 as a promoting factor of DNA repair.

### Activated AKT deubiquitinates and upregulates CRIP1 in response to DNA damage signals

**Fig. 6:**
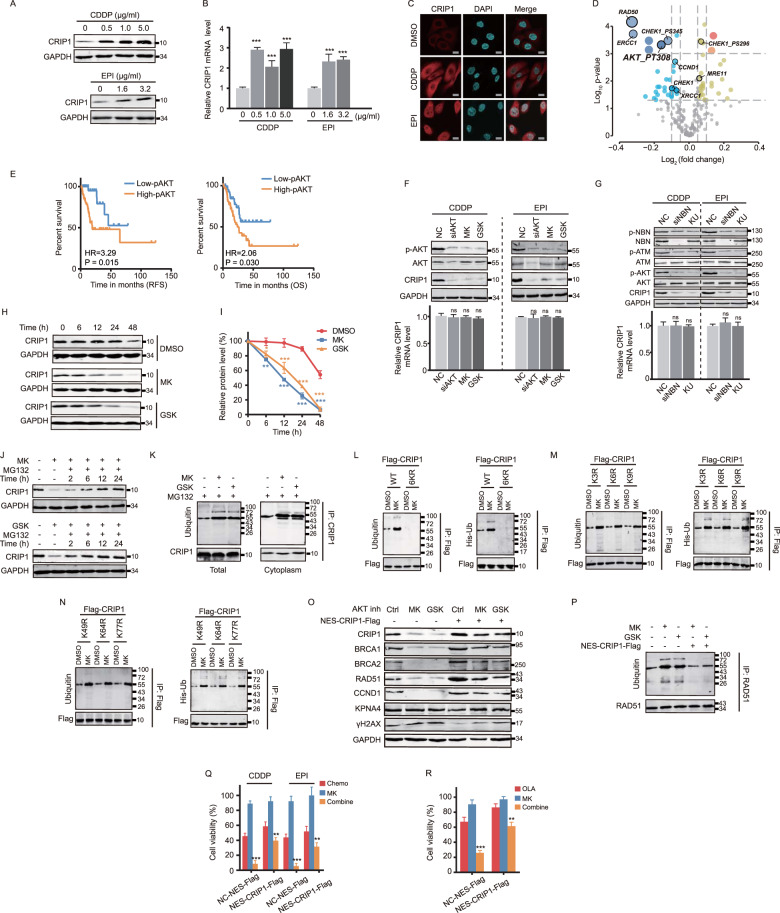
CRIP1 is upregulated by DNA damage stress via AKT activation in AGS cells. **A**, **B** Western blotting (**A**) and real-time PCR (**B**) analysis of CRIP1 expression in AGS cells treated with chemotherapeutic drugs of different drug concentration. The mean ± SD of three replicates were shown. **C** Immunofluorescence assay to detect CRIP1 expression in AGS cells treated with vehicle or chemotherapeutic drugs. Scale bar, 10 μm. **D** Volcano plot showing the molecules that is correlated with CIRP1 mRNA expression in TCGA “stomach provisional database.” The size of dots represents absolute value of log_10_(*p*-value) and the shade of color reflects the value of log_2_(fold change). **E** Kaplan–Meier curves of recurrence-free survival (RFS) and overall survival (OS) according to AKT phosphorylation levels in gastric cancer patients of the TCGA dataset. **F**–**G** Western blot and real-time PCR analysis of CRIP1 expression in AGS cells treated with vehicle (or empty vector) or AKT dephosphorylation inducers, such as siRNA-mediated knockdown (**F**), kinase inhibitors (**F**), or suppression the activation of upstream molecules (**G**) under chemotherapeutic drug stimulation. The mean ± SD of three replicates were shown. **H**–**I** Western blot analysis of CRIP1 protein level (H) and quantification of blot intensity (**I**) in AGS cells at different time point after treatment with vehicle or AKT inhibitors. Cycloheximide was added into cell culture medium simultaneously to block endogenous protein synthesis. The relative protein expression level of CRIP1 protein was determined as the relative blot intensity of CRIP1 to that of GAPDH at each time point and was set as 1 for cells at the time 0 h. The mean ± SD of three replicates of each time point were shown. **J** Western blot analysis of CRIP1 protein level in AGS cells treated with AKT inhibitors combined with vehicle or MG132. The whole-cell lysate of AGS cells without any treatment was used as a control for the expression level of CRIP1. **K** Co-immunoprecipitation analysis of CRIP1 ubiquitination level in whole-cell and cytoplasmic lysates of AGS cells treated with vehicle or AKT inhibitors. **L**–**N** Co-immunoprecipitation analysis of CRIP1 ubiquitination level in AGS cells transfected with indicated exogenous flag-tagged CRIP1 mutant constructs under stimulation of vehicle or MK2206. The flag-CRIP1 was immunoprecipitated using anti-flag and blots of endogenous (left) and exogenous (right) ubiquitination were probed with the ubiquitin antibody and the anti-his, respectively. **O** Western blot analysis of protein expressions of HR factors in AGS cells transfected with empty vector or NES-CRIP1-Flag construct under stimulation of vehicle or AKT inhibitors. **P** Co-immunoprecipitation analysis of RAD51 ubiquitination level in AGS cells transfected with empty vector or NES-CRIP1-Flag construct under stimulation of vehicle or AKT inhibitors. **Q** Cell viability of AGS cells transfected with empty vector or exogenous Flag-NES-CRIP1 construct after treatment with chemotherapeutic drug, MK2206, or chemotherapeutic durg plus MK2206 for 24 h as determined by MTT assay. The mean ± SD of five independent experiments were shown. **R** Cell viability of AGS cells transfected with control or exogenous NES-CRIP1-Flag plasmid after treatment with olaparib, MK2206, or olaparib plus MK2206 for 24 h as determined by MTT assay. The mean ± SD of five replicates were shown. DDP, cisplatin; EPI, epirubicin; OLA, Olaparib; NC, negative control. *p < 0.05; **p < 0.01; ***p < 0.001.

Although we indicated above that CRIP1 expression levels were associated with HR efficiency, however, it remained unclear whether CRIP1 itself senses DNA damage. Therefore, we analyzed the alterations in CRIP1 expression in GC cells in the presence of EPI or CDDP. CRIP1 expression was significantly elevated at both the protein and mRNA levels following exposure to chemotherapy in a dose-independent manner (Fig. 6A, B and Supplementary Fig. S6A, B). Immunofluorescence assays indicated that CRIP1 protein was upregulated in the cytoplasm and nucleus upon chemotherapy stimulation without forming foci (Fig. 6C and Supplementary Fig. S6C). This was consistent with the aforementioned finding that cytoplasmic CRIP1 was enriched in the nucleus during the response to genotoxic lesions.

To further explore the mechanism by which chemotherapeutic drugs upregulate cytoplasmic CRIP1 expression, the TCGA “stomach provisional database” was employed to identify proteins associated with CRIP1 expression via the “Enrichment” module of the cBioportal website. As a result, we identified a series of DDR-relevant molecules, whose protein expression or phosphorylation levels were significantly correlated with CRIP1 mRNA expression levels (Fig. 6D). Among these proteins, AKT phosphorylation plays an important role in maintaining cell genome stability [23] and correlates with shorter recurrence-free survival and OS in TCGA GC patients receiving chemotherapy (Fig. 6E). Thus, we determined whether CRIP1 is upregulated by AKT. Interestingly, dephosphorylation of AKT through multiple approaches (siAKT, AKT inhibitors, siNBN, ATM inhibitor, and ATR inhibitor) dramatically decreased CRIP1 protein, but not mRNA, expression levels in the presence of EPI or CDDP, implying a posttranscriptional mechanism (Fig. 6F–G and Supplementary Fig. S6D–F). However, we did not observe an alteration of the total expression or phosphorylation level of ATR, CHEK1, ATM, and CHEK2 after either siCRIP1 treatment or CRIP1 overexpression (Supplementary Fig. S6G), further suggesting that AKT activation is the upstream of CRIP1 expression regulation. Moreover, co-treatment of cycloheximide with AKT inhibitors (MK2206 or GSK690693) shortened the half-life of the CRIP1 protein and such effect was reversed by MG132 in a time-dependent manner (Fig. 6H–J and Supplementary Fig. S6H–J). Consistently, AKT inhibitors increased CRIP1 ubiquitination in both whole-cell and cytoplasmic lysates (Fig. 6K and Supplementary Fig. S6K), and the MK2206-induced CRIP1 ubiquitination (both endogenous and exdogenous his-tagged ubiquitination) was abolished when we transfected a flag-tagged plasmid containing mutations at all six potential ubiquitination sites of CRIP1 (K3, K6, K9, K49, K64, and K77; predicted by Ubisite, Ubpred, and Nextprot websites), whereas a single mutation at any of these sites did not impair AKT inhibition-induced CRIP1 ubiquitination (Fig. 6L–N and Supplementary Fig. S6L–N). Finally, we performed rescue experiments to confirm an AKT-CRIP1 axis in CRIP1-mediated DDR. Restoring cytoplasmic CRIP1 expression effectively rescued the reduction of the molecular expression of the BRCA2–RAD51 axis, increased RAD51 ubiquitination, repaired deficiencies, and enhanced drug sensitivity to chemotherapy and olaparib caused by AKT inhibition (Fig. 6O–R and Supplementary Fig. S6O, P).

### RAD51 inhibition sensitizes cells expressing high levels of CRIP1 to chemotherapy

**Fig. 7:**
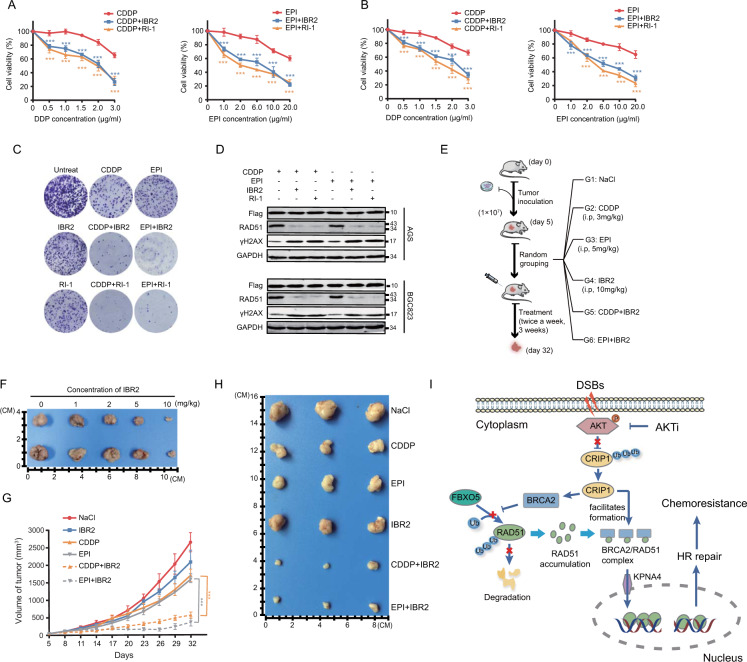
Inhibiting RAD51 restores chemosensitivity of gastric cancer cells with CRIP1 overexpression. **A**, **B** Dose–response curves of AGS cells (**A**) and BGC823 cells (**B**) treated with chemotherapeutic drugs or chemotherapeutic drugs plus RAD51 inhibitors for 24 h. The mean ± SD of five replicates were shown. **C** Colony formation ability of BGC823 cells with CRIP1-stable overexpression in the absence or presence of chemotherapeutic drugs or RAD51 inhibitors. **D** Western blot analysis of RAD51 and γH2AX protein levels in AGS and BGC823 cells treated with chemotherapeutic drugs with or without RAD51 inhibitors. **E** Schematic representation of the preclinical study design and experimental workflow. **F** Xenograft tumors of killed mice in treatment groups of different concentrations of IBR2 at the end of the toleration and dose escalation experiment. **G** Growth curves of subcutaneous xenograft tumors in different treatment groups. Each group contains three mice and *p*-values were calculated by comparing the size of xenograft tumors at the time of mice killing. **H** Xenograft tumors of killed mice in treatment groups based on different regimens. **I** The working model of the regulation of chemosensitivity and HR by CRIP1. CDDP, cisplatin; EPI, epirubicin; DSBs DNA double-strand breaks. ***p < 0.001.

Finally, we tested whether inhibition of RAD51 activity could potentially enhance chemotherapeutic efficacy in GC patients with high CRIP1 expression. The combination of RAD51 inhibitors (IBR2 and RI-1) with either CDDP or EPI resulted in a significant increase in cell death in both AGS and BGC823 cells transfected with the Flag-NES-CRIP1 plasmid (Fig. 7A, B) and in BGC823 cells stably overexpressing CRIP1 (Fig. 7C) compared with that induced by EPI or CDDP alone. Moreover, the increase in DSBs induced by the combination of chemotherapy drugs and RAD51 inhibitors was visualized as an accumulation of γH2AX in western blot assays (Fig. 7D), indicating a synergistic effect on DNA damage enhancement. Consistent with the in vitro experimental results, IBR2 (10 mg/kg) also conferred hypersensitivity to both EPI and CDDP treatment of xenograft tumors obtained using CRIP1-overexpressing GC cells (Fig. 7E–H).

## Discussion

Accumulating evidence has implicated HR efficiency in the acquisition of chemoresistance [24]. Here we identified CRIP1 as a novel regulator of HR repair and chemosensitivity in GC cells (Fig. 7I): upon DNA damage, AKT signaling is activated to stabilize CRIP1, which in turn drives RAD51 nuclear enrichment in coordination with BRCA2 to promote HR repair.

There is a consensus that the RAD51 protein is upregulated by genomic instability to ensure a sufficient nuclear accumulation level, which is a prerequisite for HR commencement [25]. However, the specific mechanisms involved in regulating RAD51 expression under genotoxic conditions remain elusive. Increased RAD51 expression reportedly does not result from gene amplification but rather from increased transcription and/or stabilization of the protein [26, 27]. Here we identified CRIP1 as a new posttranscriptional modifier of RAD51. However, in contrast to the findings of Zhang et al. [11] that CRIP1 promotes ubiquitination and degradation of Fas, exogenous overexpression of CRIP1 dramatically inhibited RAD51 ubiquitination levels, suggesting that CRIP1 itself does not possess ubiquitin ligase activity. The regulatory effect of CRIP1 on protein ubiquitination levels is indirect and mainly depends on the protease activity of ubiquitinases or deubiquitinases regulated by CRIP1. To date, several direct ubiquitinases or deubiquitinases of RAD51 have been identified. Among them, FBXO5 is one in which the protease activity is hindered by BRCA2 [16]. Our data indicate that weakening the interaction between RAD51 and FBXO5 was at least one of the mechanisms by which CRIP1, acting upstream of BRCA2, upregulated RAD51 levels upon DNA damage in GC cells. However, additional studies are needed to explore whether there are other ubiquitinases or deubiquitinases directly involved in regulating CRIP1-mediated RAD51 ubiquitination events.

Apart from adequate expression levels, the NES-masking interaction between BRCA2 and RAD51 is another important mechanism controlling the intracellular distribution of RAD51 [15]. However, how the BRCA2–RAD51 interaction is regulated following DNA damage has not been fully clarified. Luo et al. reported that ubiquitination of RAD51 hinders the RAD51–BRCA2 interaction [17]. Similarly, our study presents a parallel mode in which a CRIP1-dependent RAD51 deubiquitination modification facilitates the BRCA2–RAD51 binding following DNA damage. More importantly, we found that CRIP1 itself acted as a binding partner of the BRCA2–RAD51 complex. The CRIP1 binding region of RAD51 is also located in the core domain of RAD51 in which the NES motif is found, and the binding of both BRCA2 and CRIP1 to the RAD51 core domain were partially dependent on each other. These results indicate that CRIP1 is an essential co-factor for BRCA2 to sufficiently mask the RAD51 NES, and that CRIP1 and BRCA2 synergistically promote RAD51 binding. Further structural studies are necessary to better understand the precise underlying mechanism. Notably, our findings also revealed that chemotherapeutic drug stimulation also resulted in nuclear accumulation of CRIP1, and such subcellular translocation was blocked by silencing of either BRCA2 or RAD51. These results indicate that CRIP1 might act as a chaperone to maintain the stability of the BRCA2–RAD51 complex throughout the BRCA2-mediated RAD51 nuclear retention process. In addition, through MS screening, we identified KPNA4 as at least one of the carriers controlling the nucleo-cytoplasmic distribution of the CRIP1–BRCA2–RAD51 complex. In the future, targeting KPNA4 may be an effective strategy for enhancing chemotherapeutic benefits to GC patients.

In recent years, targeted molecular therapies have attracted widespread attention. Although several molecular drivers have been identified for GC, most failed to be translated into further clinical applications [28–34], necessitating the identification of new molecular targets with clinical transformation significance. Our study has uncovered that chemotherapy leads to AKT kinase phosphorylation, which is an upstream activator required for triggering the CRIP1-dependent HR process. AKT activation is detected in ~30% of tumor biopsies of Chinese GC patients [35], a higher rate than HER2 positivity rates (~10%–12%) [36, 37]. AKT may also be an ideal target molecule. Notably, there has already been a phase 2 clinical trial assessing the efficacy and safety of the AKT kinase inhibitor MK2206 in GC patients in second line settings [38]. Unfortunately, a negative result was yielded, suggesting that MK2206 monotherapy does not bring survival benefits to GC patients. As MK2206 has shown a synergistic effect with several commonly used chemotherapeutic agents and olaparib, combination therapy models provide a rationale for a treatment strategy that should be considered in future clinical studies on GC patients.

## Materials and methods

### Patients and tumor tissue specimens

This study was approved by the Nanfang Hospital Ethics Review Board. A total of 298 paraffin-embedded samples from patients with GC were collected. The patients were all histologically diagnosed with GC at Nanfang Hospital, Southern Medical University (Guangzhou, China). Among them, 44 patients were at stage I, 87 were at stage II, 134 were at stage III, and 33 were at stage IV. All patients underwent a radical operation (stage I–III) or a palliative surgery (stage IV) with chemotherapy (peri- or postoperative). We also downloaded two GC datasets with clinical information from TCGA (STAD, Stomach adenocarcinoma) and Gene Expression Omnibus (GSE62254).

### Cell lines

Cancer cell lines, including AGS, BGC823, HGC27, and MKN45, as well as the immortalized gastric epithelial cell line GES-1, were routinely maintained in Roswell Park Memorial Institute 1640 medium with 10% fetal bovine serum and cultured at 37 °C under 5% CO_2_. All cell lines were authenticated by the short tandem repeat profiling.

### Compounds and reagents

Cisplatin and EPI were purchased from Shanghai Yuanye Bio-Technology Co. Ltd (Shanghai, China). MK2206, LY294002, GSK690693, KU-55933, RI-1, MG132, VE-821, aphidicolin, and 3-MA were purchased from Selleck Chemicals LLC (Shanghai, China). IRB2 and cycloheximide were purchased from MedChemExpress (Shanghai, China). Thymidine were purchased from Abcam (Cambridge, UK). Lipofectamine 2000 reagent, Opti-MEM, and IP lysis buffer were purchased from Invitrogen (Shanghai, China).

### Cell transfection

Details for gene transient transfection and stable transfection are provided in Supplementary Materials and Methods. Specific siRNA sequences are shown in Supplementary Table S1.

### IHC staining

IHC staining was performed routinely as previously described [39]. The intensity of staining was scored as 0 (negative), 1 (weak), 2 (medium), or 3 (strong), whereas the extent of staining was scored as 0 (0% of cells stained), 1 (1%–25%), 2 (26%–50%), 3 (51%–75%), or 4 (76%–100%). The final protein expression score was calculated using the product of intensity and extent of staining scores.

### Western blotting, immunoprecipitation, immunofluorescence, and quantitative real-time PCR

Western blotting, immunoprecipitation, and immunofluorescence assays were performed as described previously using specific antibodies listed in Supplementary Table S2 [39, 40]. Quantitative real-time PCR was performed using the SYBR Green I Master kit (Roche, Basel, Switzerland) with a LightCycler 480 system as described previously [39, 41]. Primer sequences involved in the present study are listed in Supplementary Table S3.

### EdU, MTT, and clonogenic assays

Cell survival and proliferation were measured using EdU, MTT, and clonogenic assays, as previously described [40, 41].

### Flow cytometry

Flow cytometry analysis was performed using Annexin V-FITC/PI Apoptosis kits (Keygen Biotech, Nanjing, China) according to the manufacturer’s instructions. Details are provided in Supplementary Materials and Methods.

### HR assay

The DR-GFP reporter system containing an upstream GFP gene with an I-SceI recognition site (SceGFP) and a downstream internal GFP repeat was utilized. Details are provided in Supplementary Materials and Methods.

### Comet assay

The comet assay was used to measure DNA strand breaks in single cells. The assay was performed using the Comet Assay for DNA Damage Detection Kit (KeyGen, Biotechnology, China) according to the manufacturer’s instructions. Details are provided in Supplementary Materials and Methods.

### Liquid chromatography–MS/MS

Cell lysate preparation for liquid chromatography–MS/MS analysis was conducted in the same manner as that described for immunoprecipitation assays [40, 42]. Details are provided in Supplementary Materials and Methods.

### Animal experiments

All animal experiments were approved by the Nanfang Hospital Animal Care and Use Committee, and followed the National Guidelines for Animal Experimentation. Details are provided in Supplementary Materials and Methods.

### Statistical analyses

Details are provided in Supplementary Materials and Methods.

## Supplementary information

Supplemental Figure 1

Supplemental Figure 2

Supplemental Figure 3

Supplemental Figure 4

Supplemental Figure 5

Supplemental Figure 6

Supplemental Figure Legends

Supplemental Table 1

Supplemental Table 2

Supplemental Table 3

Supplemental Materials and Methods

## Data Availability

The authors declare that all data supporting the findings of this study are available within the article and its Supplementary Information files. The public data used in this study are available at GSE62254, TCGA STAD dataset (http://www.cbioportal.org/), and Oncomine dataset (https://www.oncomine.org/resource/main.html).
