# Supplementary figures and images for: CRIP1 cooperates with BRCA2 to drive the nuclear enrichment of RAD51 and to facilitate homologous repair upon DNA damage induced by chemotherapy
Source: Oncogene. 2021 Jul 14;40(34):5342–55. doi: 10.1038/s41388-021-01932-0 (PMC8390368; doi:10.1038/s41388-021-01932-0)

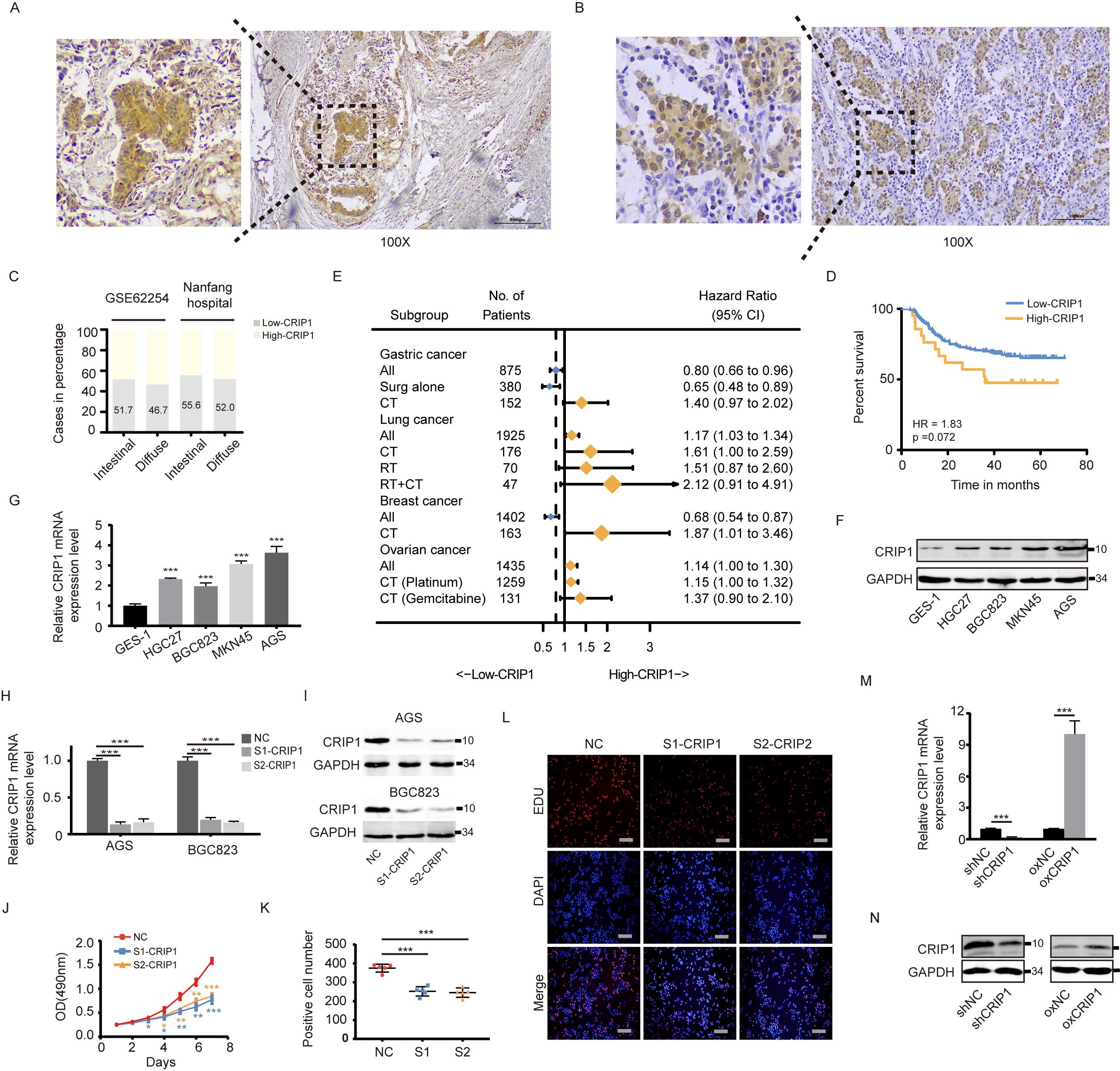

Supplement: Supplementary file 1 — Supplemental Figure 1 [file 41388_2021_1932_MOESM1_ESM.tif]

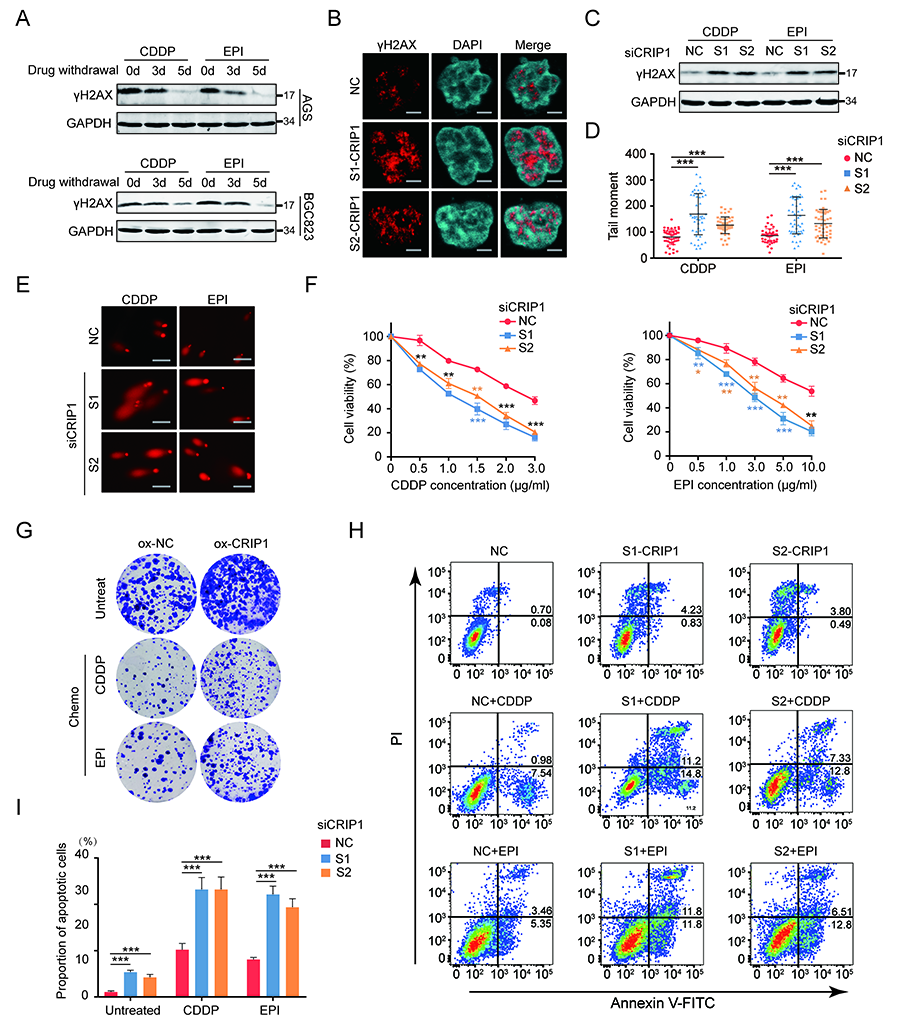

Supplement: Supplementary file 2 — Supplemental Figure 2 [file 41388_2021_1932_MOESM2_ESM.tif]

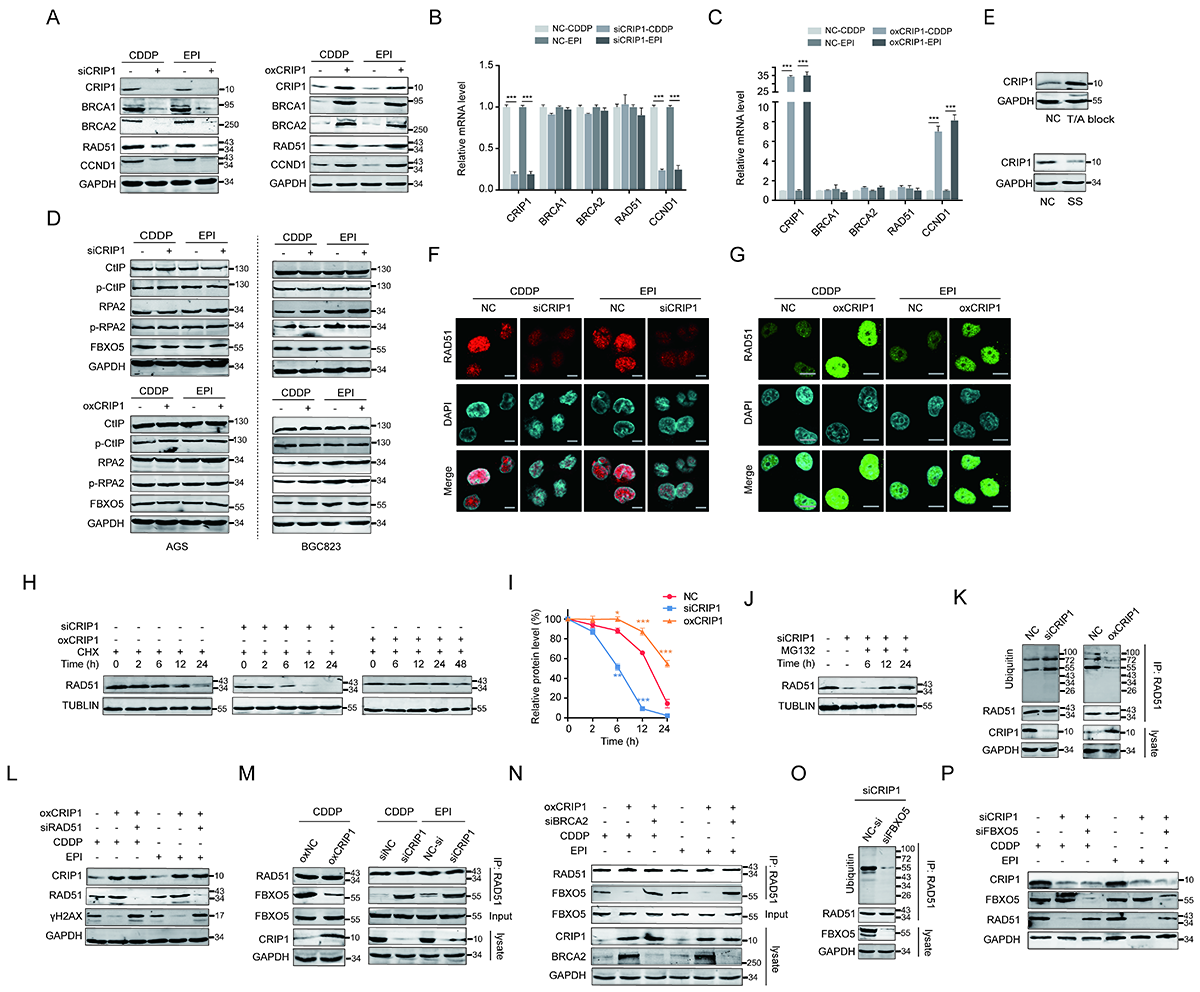

Supplement: Supplementary file 3 — Supplemental Figure 3 [file 41388_2021_1932_MOESM3_ESM.tif]

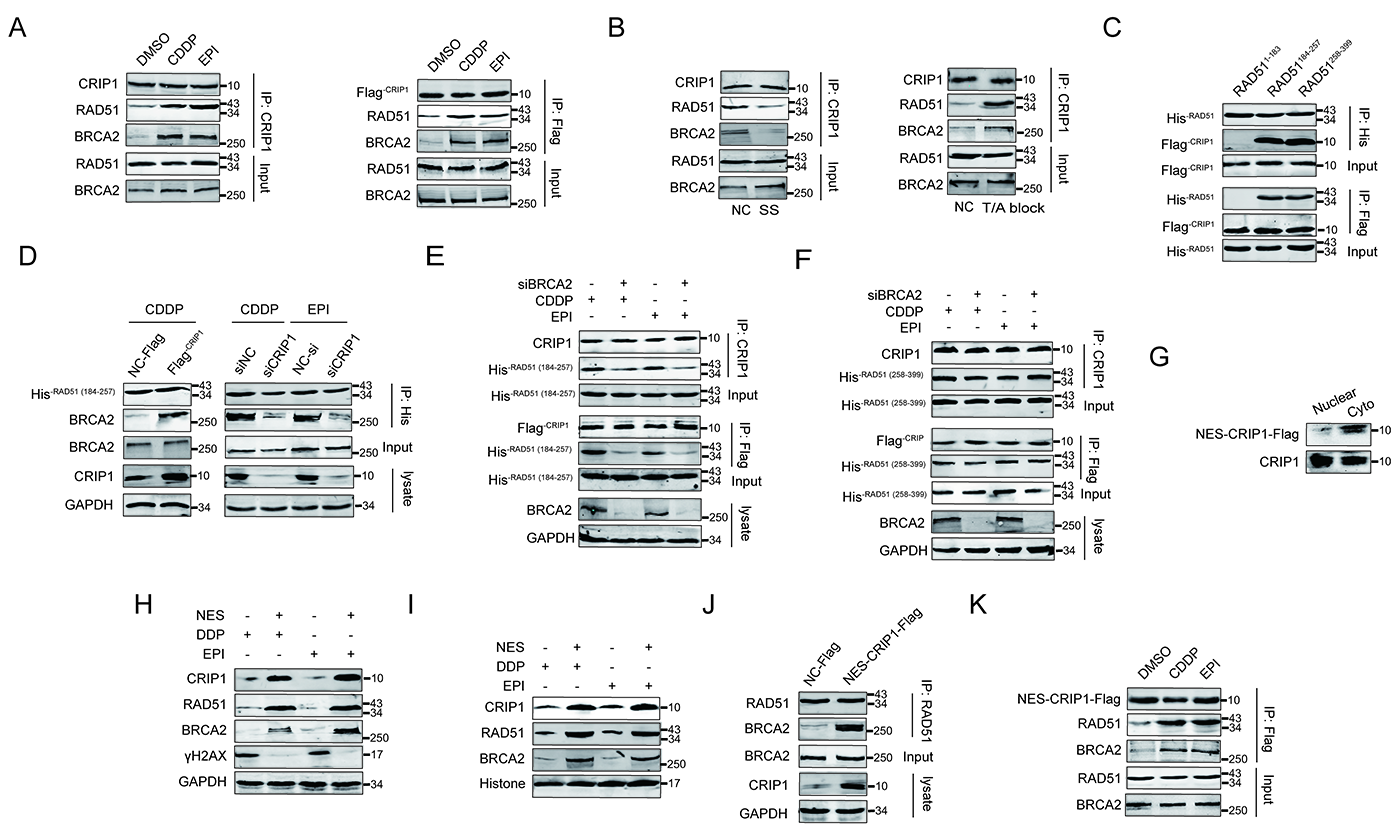

Supplement: Supplementary file 4 — Supplemental Figure 4 [file 41388_2021_1932_MOESM4_ESM.tif]

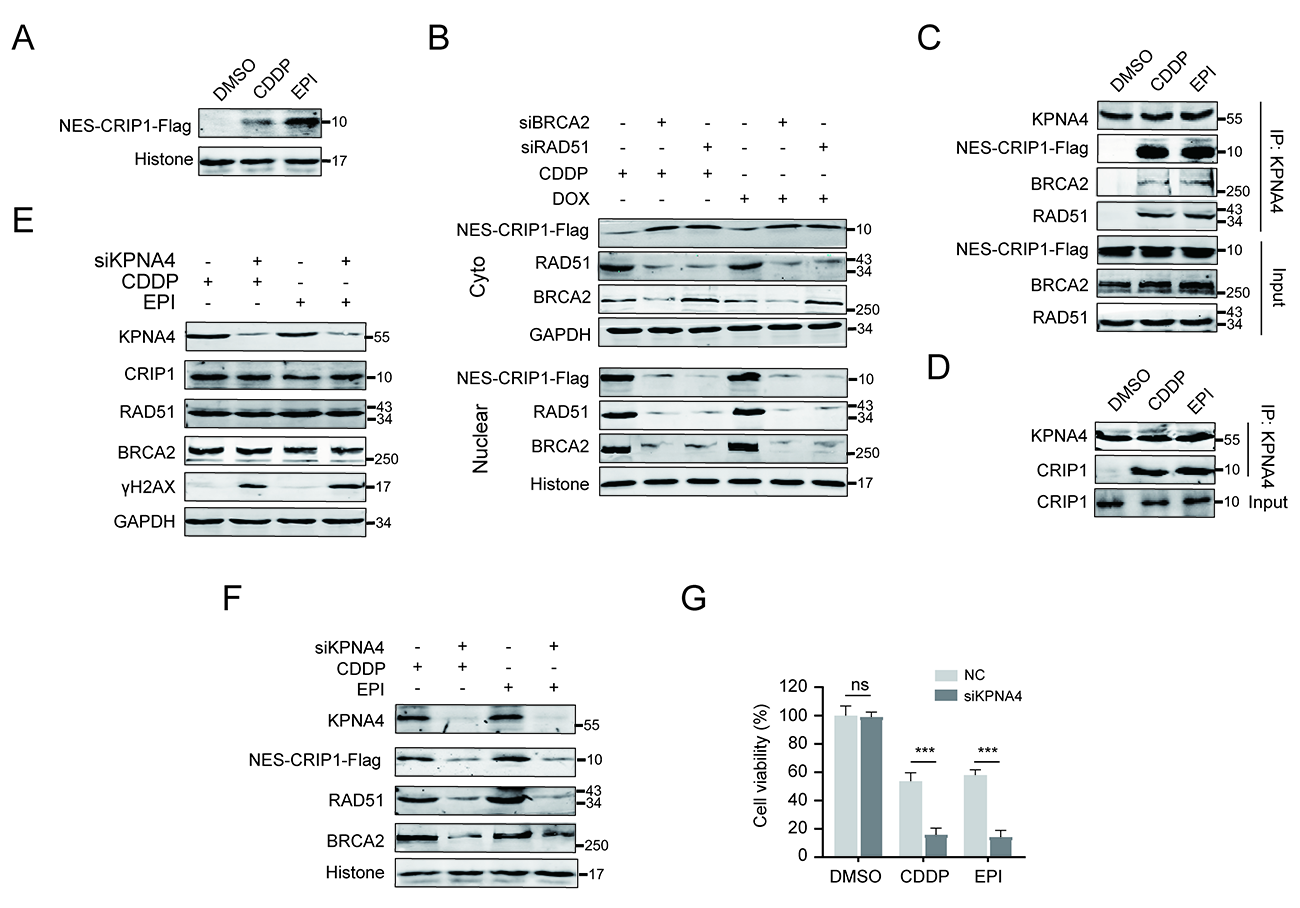

Supplement: Supplementary file 5 — Supplemental Figure 5 [file 41388_2021_1932_MOESM5_ESM.tif]

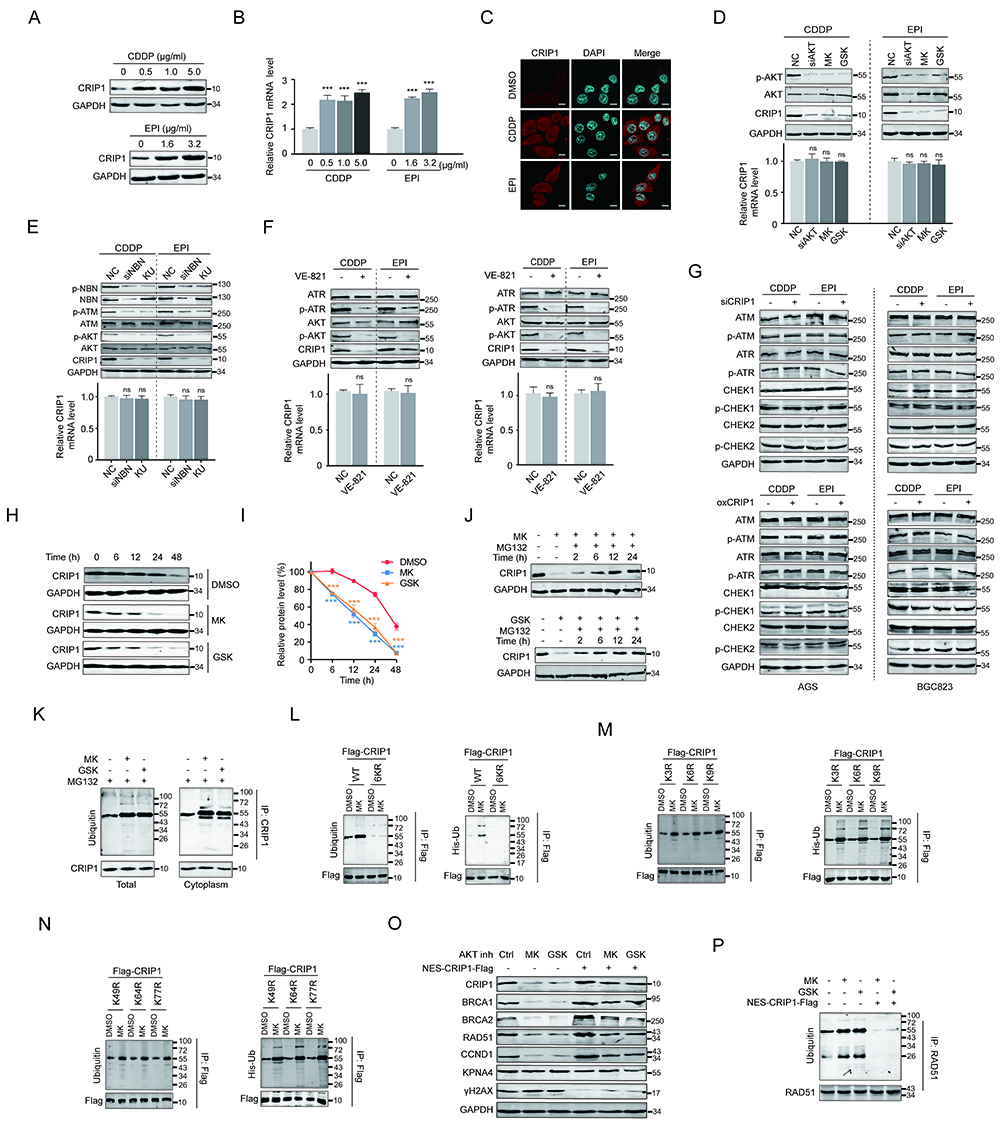

Supplement: Supplementary file 6 — Supplemental Figure 6 [file 41388_2021_1932_MOESM6_ESM.tif]
