# Supplementary material for: CRIP1 cooperates with BRCA2 to drive the nuclear enrichment of RAD51 and to facilitate homologous repair upon DNA damage induced by chemotherapy
Source: Oncogene. 2021 Jul 14;40(34):5342–55. doi: 10.1038/s41388-021-01932-0 (PMC8390368; doi:10.1038/s41388-021-01932-0)
Supplement: Supplementary file 7 — Supplemental Figure Legends [file 41388_2021_1932_MOESM7_ESM.docx]

**Figure S1.** Effects of CRIP1 on the progression of gastric tumors. (A-B) Representative micrographs of CRIP1 protein expression in non-metastatic (A) and metastatic GC tissues (B), as detected by immunohistochemistry; Scale, 100μm. (C) Bar charts summarizing proportions of patients with low CRIP1 expression within groups categorized by Lauren subtype in the GSE62254 dataset (CRIP1 mRNA expession) and in the cohort of Nanfang hospital (CRIP1 protein expression). The chi square tests were used for statistical comparisons; (D) Kaplan-Meier curves of disease-free survival according to CRIP1 expression groups in GSE62254 datasets; (E) Forest plots of the associations between CRIP1 expression and overall survival in various subgroups of “Kaplan-Meier plotter” online database. Unadjusted HRs (boxes) and 95% confidence intervals (horizontal lines) were depicted; (F-G) Western blot (F) and real-time PCR (G) analysis of the CRIP1 expression in GES-1 and four GC cell lines. The mean ± SD of three replicates were shown; (H-I) Efficiency of siRNA sequences against CRIP1 was evaluated by real-time PCR (H) and western blot analysis (I). The mean ± SD of three replicates were shown; (J) Proliferation of BGC823 cell transfected with empty vector or CRIP1 siRNA, as determined by MTT assay. The mean ± SD of five replicates of each time point were shown; (K-L) Proliferation of BGC823 cell transfected with empty vector or CRIP1 siRNA, as determined by EdU assay (L) and quantification of the number of positive cell number (K). The mean ± SD of three replicates were shown. Scale, 100μm; (M-N) Efficiency of stable CRIP1 knockdown and overexpressing as confirmed by real-time PCR (M) and western blot analysis (N). The mean ± SD of three replicates were shown. *HR, hazard ratio; CT, chemotherapy; RT, radiotherapy; CI, confidence interval; NC, negative control. *, p<0.05; **, p<0.01; ***, p<0.001.*

**Figure S2.** CRIP1 deletion inhibits DNA repair and increases susceptibility to chemotherapy in BGC823 cells. (A) Western blot analysis of γH2AX protein level in BGC823 cells at different time point after cisplatin (1μg/ml, treat for 24 hours before withdrawal) or epirubicin (1μg/ml, treat for 24 hours before withdrawal) withdrawal; (B) Representative images of γH2AX staining of BGC823 cells transfected with empty vector or CRIP1 siRNA after cisplatin withdrawal. Scale, 2.5μm; (C) Western blot analysis of γH2AX protein level in BGC823 cells transfected with empty vector or CRIP1 siRNA after cisplatin or epirubicin withdrawal; (D-E) Neutral comet assay measure of DNA damage in the BGC823 cells transfected with empty vector or CRIP1 siRNA under stimulation of cisplatin or epirubicin. Representative image (E) and quantification of tail moments (D) were shown. 50 replicates were used. Scale, 100μm; (F) Dose-response curves of BGC823 cells transfected with empty vector or CRIP1 siRNA after treatment with cisplatin or epirubicin for 24 h. The mean ± SD of five replicates of each time point were shown; (G) Colony formation ability of CRIP1 stable overexpressing and corresponding control BGC823 cells with or without chemotherapeutic drug treatment; (H-I) Flow cytometry analysis of apoptosis of AGS cells transfected with empty vector or CRIP1 siRNA under stimulation of vehicle or chemotherapeutic drugs. The sum of Q2 and Q3 represents the total percentage of early and late apoptotic cells (H). The proportions of apoptotic cells were displayed by the bar chart (I). The mean ± SD of three replicates were shown. C*DDP,* c*isplatin; EPI, epirubicin; NC, negative control; ox-CRIP1, CRIP1 overexpression.* **, p<0.05; **, p<0.01; ***, p<0.001.*

**Figure S3.** CRIP1 is required for homologous recombination (HR) repair in BGC823 cells. (A) Western blot analysis of the protein level of molecules involved in regulating BRCA2-RAD51 axis in CRIP1 knockdown (left) or overexpressing (right) BGC823 cells as compared to the control group after cisplatin or epirubicin treatment; (B-C) Real-time PCR analysis of the mRNA level of molecules involved in BRCA2-RAD51 axis in CRIP1 silencing (B) or overexpressing (C) BGC823 cells as compared to the control group after cisplatin or epirubicin treatment. The mean ± SD of three replicates were shown; (D) Western blot analysis of CtIP, CtIP, RPA, p-RPA and FBXO5 expression levels in CRIP1 knockdown or overexpressing BGC823 cells as compared to the control group after cisplatin or epirubicin treatment; (E) Western blot analysis of CRIP1 expression in BGC823 cells treated with thymidine/aphidicolin block (upper) or serum starvation (down); (F-G) Immunofluorescence assays of RAD51 expression and foci formation in CRIP1 silencing (F) or overexpressing (G) BGC823 cells as compared to the control group after cisplatin or epirubicin treatment. Scale, 10 μm; (H-I) Western blot analysis of RAD51 protein level (H) and quantification of blot intensity (I) in BGCC823 cells transfected with empty vector, CRIP1 siRNA or CRIP1 plasmid at different time point after cycloheximide treatment. The western blot assays for the protein samples in the siCRIP1, oxCRIP1, and empty vector groups were performed separately in different gels. The loading amount and exposure intensity for the protein sample of each group were adjusted to ensure clarity of the blots. At 48h post transfection, cycloheximide was added into cell culture medium to block endogenous protein synthesis. The relative protein expression level of RAD51 protein was determined as the relative blot intensity of RAD51 to that of TUBLIN at each time point, and was set as 1 for cells at the time-0h. The mean ± SD of three replicates of each time point were shown; (J) Western blot analysis of RAD51 protein level in CRIP1-knockdown BGC823 cells treated with vehicle or MG132. The whole-cell lysate of BGC823 cells transfected with empty vector was used as a control for the expression level of RAD51; (K) Co-immunoprecipitation analysis of RAD51 ubiquitination level in CRIP1-knockdown or overexpressing BGC823 cells as compared to the control group. RAD51 was immunoprecipitated and blots of endogenous ubquitination were probed with the ubiquitin antibody; (L) Western blot analysis of CRIP1, RAD51, and γH2AX protein levels in BGC823 cells transfected with CRIP1 plasmid alone or co-transfected with CRIP1 plasmid and RAD51 siRNA under chemotherapeutic drug stimulation; (M) Co-immunoprecipitation analysis of the interaction between RAD51 and FBXO5 in CRIP1-knockdown or overexpressing BGC823 cells as compared to the control group under chemotherapeutic drug stimulation; (N) Co-immunoprecipitation analysis of the interaction between RAD51 and FBXO5 in BGC823 cells transfected with CRIP1 plasmid alone or co-transfected with CRIP1 plasmid and BRCA2 siRNA under chemotherapeutic drug stimulation; (O) Co-immunoprecipitation analysis of RAD51 ubiquitination level in BGC823 cells co-transfected with CRIP1 siRNA and FBXO5 siRNA as compared to the control group. RAD51 was immunoprecipitated and blots of endogenous ubquitination were probed with the ubiquitin antibody; (P) Western blot analysis of CRIP1, RAD51, and FBXO5 protein levels in chemotherapeutic drug-treated BGC823 cells which were transfected with CRIP1 siRNA alone or co-transfected with CRIP1 siRNA and FBXO5 siRNA as compared to the control group. C*DDP,* c*isplatin; EPI, epirubicin; NC, negative control; SS,* serum starvation; T/A, thymidine/aphidicolin; *ox-CRIP1, CRIP1 overexpression. *, p<0.05; **, p<0.01; ***, p<0.001.*

**Figure S4.** CRIP1 interacts with BRCA2-RAD51 complex in BGC823 cells. (A) Co-immunoprecipitation analysis of interactions of endogenous CRIP1 (left) and exogenous flag-tagged CRIP1 (right) with RAD51 and BRCA2 in BGC823 cells treated with vehicle or chemotherapeutic drugs; (B) Co-immunoprecipitation analysis of interactions of CRIP1 with RAD51 and BRCA2 in BGC823 cells treated with serum starvation (left) or thymidine/aphidicolin block (right); (C) Co-immunoprecipitation analysis of interactions of flag-tagged CRIP1 with his-tagged RAD51 fragments in BGC823 cells; (D) Co-immunoprecipitation analysis of the interactions between RAD51^184-257^ and BRCA2 in BGC823 cells transfected with flag-tagged CRIP1 or CRIP1 siRNA as compared to the control group under chemotherapeutic drug stimulation; (E-F) Co-immunoprecipitation analysis of interactions of endogenous CRIP1 (upper) and exogenous flag-tagged CRIP1 (down) with RAD51^184-257^ (E) and RAD51^258-399^ (F) in BGC823 cells transfected with empty vector or BRCA2 siRNA under chemotherapeutic drug stimulation; (G) Western blot assay to detect subcellular localization of NES-CRIP1-Flag protein in BGC823 cells; (H-I) Western blot analysis of HR-related proteins in cytoplasm (H) and nucleus (I) of BGC823 cells transfected with empty vector or NES-CRIP1-Flag construction; (J) Co-immunoprecipitation analysis of the interaction between RAD51 and BRCA2 in BGC823 cells transfected with empty vector or NES-CRIP1-Flag; (K) Co-immunoprecipitation analysis of the interactions between NES-CRIP1-Flag and BRCA2-RAD51 complex in BGC823 cells treated with vehicle or chemotherapeutic drugs. *DMSO, dimethyl sulphoxide; NES, nuclear export signal;* C*DDP, cisplatin; EPI, epirubicin; CI, confidence interval; NC, negative control; SS,* serum starvation; T/A, thymidine/aphidicolin*.*

**Figure S5.** KPNA4 assists nuclear translocation of CRIP1/BRCA2/RAD51 complex during DNA damage response in BGC823 cells. (A) Western blot analysis of protein level of NES-CRIP1-Flag in the nucleus of BGC823 cells treated with vehicle or chemotherapeutic drugs; (B) Western blot analysis of NES-CRIP1-Flag, RAD51 and BRCA2 protein levels in cell cytoplasm and nucleus in BGC823 cells transfected with BRCA2 siRNA or RAD51 siRNA as compared to the control group under chemotherapeutic drug stimulation; (C) Co-immunoprecipitation analysis of the interactions between KPNA4, NES-CRIP1-Flag, and HR-related proteins in BGC823 cells treated with vehicle or chemotherapeutic drugs; (D) Co-immunoprecipitation analysis of the interaction between KPNA4 and endogenous CRIP1 in BGC823 cells treated with vehicle or chemotherapeutic drugs; (E-F) Western blot analysis of HR-related proteins in cytoplasm (E) and nucleus (F) of BGC823 cells transfected with empty vector or KPNA4 siRNA under chemotherapeutic drug stimulation; (G) Cell viability of BGC823 cells transfected with empty vector or KPNA4 siRNA after treatment with vehicle or chemotherapeutic drugs for 24 h as determined by MTT assay. The mean ± SD of five replicates of each time point were shown. *NES, nuclear export signal; DMSO, dimethyl sulphoxide; DDP,* c*isplatin; EPI, epirubicin; NC, negative control.* ****, p<0.001.*

**Figure S6.** CRIP1 is upregulated by DNA damage stress via AKT in BGC823 cells. (A-B) Western blot (A) and real-time PCR (B) analysis of CRIP1 expression in BGC823 cells treated with chemotherapeutic drugs of different drug concentration. The mean ± SD of three replicates were shown; (C) Immunofluorescence assay to detect CRIP1 expression in BGC823 cells treated with vehicle or chemotherapeutic drugs. Scale, 10μm; (D-E) Western blot and real-time PCR analysis of CRIP1 expression in BGC823 cells treated with vehicle or AKT dephosphorylation inducers, such as siRNA-mediated knockdown (D), kinase inhibitors (D) or suppression the activation of upstream molecules (E) under chemotherapeutic drug stimulation. The mean ± SD of three replicates were shown; (F) Western blot and real-time PCR analysis of CRIP1 expression in AGS (left) and BGC823 (right) cells treated with vehicle or the ATR inhibitor under chemotherapeutic drug stimulation. The mean ± SD of three replicates were shown; (G) Western blot analysis of total and phosphorylation level of ATM, ATR, CHEK1, CHEK2 expression in AGS and BGC823 cells transfected with empty vector, CRIP1 siRNA, or CRIP1 plasmid under chemotherapeutic drug stimulation; (H-I) Western blot analysis of CRIP1 protein level (H) and quantification of blot intensity (I) in BGC823 cells at different time point after treatment with vehicle or AKT inhibitors. Cycloheximide was added into cell culture medium simultaneously to block endogenous protein synthesis. The relative protein expression level of CRIP1 protein was determined as the relative blot intensity of CRIP1 to that of GAPDH at each time point, and was set as 1 for cells at the time-0h; (J) Western blot analysis of CRIP1 protein level in BGC823 cells treated with MK2206 combined with vehicle or MG132. The whole-cell lysate of BGC823 cells without any treatment was used as a control for the expression level of CRIP1. The mean ± SD of three replicates of each time point were shown; (K) Co-immunoprecipitation analysis of CRIP1 ubiquitination level in whole-cell and cytoplasmic lysates of BGC823 cells treated with vehicle or AKT inhibitors; (L-N) Co-immunoprecipitation analysis of CRIP1 ubiquitination level in BGC823 cells transfected with indicated exogenous flag-tagged CRIP1 mutant constructs under stimulation of vehicle or MK2206. The flag-CRIP1 was immunoprecipitated using anti-flag and blots of endogenous (left) and exogenous (right) ubiquitination were probed with the ubiquitin antibody and the anti-his respectively; (O) Western blot analysis of protein expressions of HR factors in BGC823 cells transfected with empty vector or NES-CRIP1-Flag construct under stimulation of vehicle or AKT inhibitors; (P) Co-immunoprecipitation analysis of RAD51 ubiquitination level in BGC823 cells transfected with empty vector or NES-CRIP1-Flag construct under stimulation of vehicle or AKT inhibitors. *DDP,* c*isplatin; EPI, epirubicin; OLA, Olaparib; NC, negative control.* **, p<0.05; **, p<0.01; ***, p<0.001.*
