# Supplementary material for: CRIP1 cooperates with BRCA2 to drive the nuclear enrichment of RAD51 and to facilitate homologous repair upon DNA damage induced by chemotherapy
Source: Oncogene. 2021 Jul 14;40(34):5342–55. doi: 10.1038/s41388-021-01932-0 (PMC8390368; doi:10.1038/s41388-021-01932-0)
Supplement: Supplementary file 8 — Supplemental Table 1 [file 41388_2021_1932_MOESM8_ESM.doc]

**Supplement Table 1 Sequences of siRNA**

| **Gene name** |  | **Primer sequences(5'-3')** |
| --- | --- | --- |
| CRIP1 | S1 | GCAACAAGGAGGUGUACUUTT  AAGUACACCUCCUUGUUGCTT |
|  | S2 | CCGAGAGCCACACUUUCAATT  UUGAAAGUGUGGCUCUCGGTT |
|  | S3 | GCCUGAAGUGCGAGAAAUGTT  CAUUUCUCGCACUUCAGGCTT |
|  | S4 | GCAAACCCUACUGCAACCATT  UGGUUGCAGUAGGGUUUGCTT |
| KPNA4 | S1 | GACAACGAAAUGAAGUUGUTT  ACAACUUCAUUUCGUUGUCTT |
|  | S2 | CAACUUAUGUCGCCACAAATT  UUUGUGGCGACAUAAGUUGTT |
|  | S3 | GGCCAUAAGUAACUUAACATT  UGUUAAGUUACUUAUGGCCTT |
| BRCA2 | S1 | GCAGAAGAAUCUGAACAUATT  UAUGUUCAGAUUCUUCUGCTT |
|  | S2 | GCAACCCAAGUGUCAAUUATT  UAAUUGACACUUGGGUUGCTT |
|  | S3 | GCCUUGGAUUUCUUGAGUATT  UACUCAAGAAAUCCAAGGCTT |
| RAD51 | S1 | GCAACUGAAUUCCACCAAATT  UUUGGUGGAAUUCAGUUGCTT |
|  | S2 | GCAGUGAUGUCCUGGAUAATT  UUAUCCAGGACAUCACUGCTT |
|  | S3 | GGGUGGAGGUGAAGGAAAGTT  CUUUCCUUCACCUCCACCCTT |
| NBN | S1 | GCUCCAAAGGCAAGGUCUUTT  AAGACCUUGCCUUUGGAGCTT |
|  | S2 | CCAAUUGUAAAGCCAGAAUTT  AUUCUGGCUUUACAAUUGGTT |
|  | S3 | GCCAAGGAUGGAUAUAGAATT  UUCUAUAUCCAUCCUUGGCTT |
| BRCA1 | S1 | CCACCUAAUUGUACUGAAUTT  AUUCAGUACAAUUAGGUGGTT |
|  | S2 | GCAGUGAAUUGGAAGACUUTT  AAGUCUUCCAAUUCACUGCTT |
|  | S3 | GCUAGAAAUCUGUUGCUAUTT  AUAGCAACAGAUUUCUAGCTT |
| FXBO5 | S1 | UUACUGAUCAUGAAUGUUA  TTGCAGGTGAATTACAGCGA |
|  | S2 | CGAAGUGUCUCUGUAAUUA  TTCTTCTTTACAACTATCCGT |
|  | S3 | UGUAUUGGGUCACCGAUUG AAACCTCAGGATATACACCG |
