# Supplementary material for: CRIP1 cooperates with BRCA2 to drive the nuclear enrichment of RAD51 and to facilitate homologous repair upon DNA damage induced by chemotherapy
Source: Oncogene. 2021 Jul 14;40(34):5342–55. doi: 10.1038/s41388-021-01932-0 (PMC8390368; doi:10.1038/s41388-021-01932-0)
Supplement: Supplementary file 9 — Supplemental Table 2 [file 41388_2021_1932_MOESM9_ESM.doc]

**Supplement Table 2 Antibodies**

| **Antibodies** | **SOURCE** | **Cat. No.** |
| --- | --- | --- |
| CRIP1 | ABclonal | A7548 |
| CRIP1 | Proteintech | 15349-1-AP |
| CRIP1 | Abcam | AB211631 |
| γH2AX (Ser-139) | Cell Signaling Technology | 5438S |
| Phospho-AKT-Thr308 | Cell Signaling Technology | 13038S |
| AKT1 | ABclonal | A7548 |
| Phospho-ATM-S1981 | Abcam | AB81292 |
| ATM | Abcam | AB32420 |
| Phospho- NBS1-S343 | Abcam | Ab109453 |
| NBS1 | Abcam | Ab32074 |
| RPA2 | ABclonal | A2189 |
| Phospho-RPA2-S4/S8 | ABclonal | AP1102 |
| BRCA1 | Cell Signaling Technology | 10823S |
| BRCA2 | Cell Signaling Technology | 10741S |
| RAD51 | Abcam | Ab183029 |
| RAD51 | ABclonal | A2829 |
| CCND1 | ABclonal | A11310 |
| Phospho-CtIP-S738 | Abcam | AB195291 |
| Anti-CtIP | Abcam | AB117722 |
| XRCC5 | ABclonal | A5862 |
| XRCC6 | ABclonal | A7330 |
| KPNA4 | ABclonal | A8347 |
| Flag-tag（MA4） | Beijing Ray Antibody | RM1002 |
| His-tag（MC10） | Beijing Ray Antibody | RM1001 |
| Flag-Tag | ABclonal | AE005 |
| Anti-Ubiquitin | Abcam | Ab134953 |
| GAPDH | Proteintech | 60004-1-Ig |
| Tublin | Proteintech | YT5843 |
| anti-Histone H3 | Abcam | AB1791 |
| FBXO5 | Proteintech | 10872-1-AP |
| Phospho-ATR-S428 | Abcam | AB178407 |
| ATR | Immunoway | YT0416 |
| Phospho-Chk1-S345 | ABclonal | AP0578 |
| Chk1 | ABclonal | A7653 |
| Phospho-Chk2-T68 | ABclonal | AP0590 |
| Chk2 | ABclonal | A7653 |
