# Supplementary material for: CRIP1 cooperates with BRCA2 to drive the nuclear enrichment of RAD51 and to facilitate homologous repair upon DNA damage induced by chemotherapy
Source: Oncogene. 2021 Jul 14;40(34):5342–55. doi: 10.1038/s41388-021-01932-0 (PMC8390368; doi:10.1038/s41388-021-01932-0)
Supplement: Supplementary file 10 — Supplemental Table 3 [file 41388_2021_1932_MOESM10_ESM.doc]

**Supplement Table 3 Primer sequences**

| **Gene name** | **Primer sequences(5'-3')** | **Primer length（bp）** |
| --- | --- | --- |
| CRIP1 | Forward: 5'-GCAACAAGGAGGTGTACTTCG-3' | 21 |
|  | Reverse: 5'-CACATTTCTCG ACTTCAGG-3' | 20 |
| GAPDH | Forward:5'-GGAGCGAGATCCCTCCAAAAT-3' | 21 |
|  | Reverse:5'-GGCTGTTGTCATACTTCTCATGG-3' | 23 |
| RAD51 | Forward: 5'-TCTCTGGCAGTGATGTCCTGGA-3' | 22 |
|  | Reverse: 5'-TAAAGGGCGGTGGCACTGTCTA-3' | 22 |
| BRCA2 | Forward: 5'-ACCCAGCTTACCTTGAGGGTTATTT-3' | 25 |
|  | Reverse: 5'-AATACGCAACTTCCACACGGTTG-3' | 23 |
| BRCA1 | Forward: 5'-TTGCAGTGTGGGAGATCAAG-3' | 20 |
|  | Reverse: 5'-CGCTTCTCAGTGGTGTTCAA-3' | 20 |
| XRCC5 | Forward: 5'-TGACTTCCTGGATGCACTAATCGT-3' | 24 |
|  | Reverse: 5'-TTGGAGCCAATGGTCAGTCG-3' | 20 |
| XRCC6 | Forward: 5'-CGATAATGAAGGTTCTGGAAG-3' | 21 |
|  | Reverse: 5'-CTGGAAGTGCTTGGTGAG-3' | 18 |
| CCND1 | Forward: 5'-GAAGCCCTGCTGGAGTCA-3' | 18 |
|  | Reverse: 5'-CCAGGTCCACCTCCTCCT-3' | 18 |
| AKT1 | Forward: 5'-TCTATGGCGCTGAGATTGTG-3' | 20 |
|  | Reverse: 5'-CTTAATGTGCCCGTCCTTGT-3' | 20 |
| FBXO5 | Forward: 5'-CTGCAAACGAGAAGGCTGTG-3' | 20 |
|  | Reverse: 5'-ACTGGCTTTGAGGAGCTTGC-3 | 20 |
