# Supplementary material for: CRIP1 cooperates with BRCA2 to drive the nuclear enrichment of RAD51 and to facilitate homologous repair upon DNA damage induced by chemotherapy
Source: Oncogene. 2021 Jul 14;40(34):5342–55. doi: 10.1038/s41388-021-01932-0 (PMC8390368; doi:10.1038/s41388-021-01932-0)
Supplement: Supplementary file 11 — Supplemental Materials and Methods [file 41388_2021_1932_MOESM11_ESM.docx]

**Supplementary Materials and Methods**

**Cell transfection**

For RNA interference, GC cells were seeded in 6-well plates until 30%–40% density was reached. Thereafter, 50 nM siRNA (GEMA, Suzhou, China), 5 μL Lipofectamine 2000 transfection reagent, and Opti-MEM were transfected into the cells according to the manufacturer’s instructions. A lentiviral vector encoding CRIP1 or sh-CRIP1 was constructed by inserting full-length CRIP1 or a small interfering RNA, respectively, into the AgeI/EcoRI site of the corresponding viral vector. The viral vector was transduced into GC cells for 2 d. After screening with puromycin, stable expression clones were selected. For plasmid transfection, cells were grown to a 60%–70% density in 6-cm cell-culture dishes. Plasmid (7 μg; GeneChem, Shanghai, China) and Lipofectamine 2000 transfection reagent (7 μL) were transfected into cells.

**Flow cytometry**

Treated cells were digested and resuspended, washed thrice with 10× binding buffer, and stained with Annexin V-FITC and PI. Finally, the samples were analyzed using flow cytometry (BD Biosciences, San Jose, CA, USA).

**Homologous recombination assay**

A total of 2.5 x 10^5^ cells were electroporated with the pDR-GFP plasmid (3 μg, #26475, Addgene, Watertown, MA, USA) and selected with puromycin. The puromycin-resistant colonies were continuously transduced for RNA interference (negative control or siCRIP1, 5 μL) for 24 h. Cells were then electroporated with pCBASceI (an I-SceI endonuclease expression vector with a mammalian promoter to introduce a DSB at a genomic I-SceI site, 3 μg, #26477, Addgene, Watertown, MA, USA) to generate a DSB. After 48 h, the cells were analyzed using flow cytometry to measure the percentages of GFP-positive cells using the FlowJo software. The percentage of GFP-positive cells reflects the number of cells in which DSB repair occurred via the HR pathway. The reporter assays were independently performed three times, each time using biological triplicate samples.

**Comet assay**

Briefly, cells were harvested in ice cold PBS and mixed with 0.5% low-melting agarose, which was then solidified on a slide. Thereafter, the cells were subjected to cell lysis, DNA unwinding, electrophoresis, neutralization, staining, and analysis via fluorescence confocal microscopy using an Olympus FV1000 microscope (Olympus, Tokyo, Japan). Tail length was measured using comet assay software.

**Liquid chromatography-tandem mass spectrometry (LC-MS/MS)**

In shortly, the precipitate obtained in IP experiment was reduced by final concentration of 10 mM DTT and the sample was incubated at 56°C for 30 min. The sample was then alkylated by adding 500 mM IAA to obtain a final concentration of 50 mM followed by incubation at room temperature for 30 min in the dark. Finally, sequencing grade trypsin was added at a trypsin: protein ratio of 1:100 (w/w), and the sample was incubated at 37°C for 16 h. The digested peptides were collected in accordance with a standard protocol for filter-aided sample preparation (FASP). Finally, the concentrated peptides were desalted through a C18 column according to the manufacturer’s protocol, and were analyzed using EASY-nLC1200 connected to an Orbitrap fusion mass spectrometer (Thermo Scientific, Waltham, MA, USA) based data dependent acquiring (DDA) strategy. The acquired DDA data were searched by MaxQuant based on the human reviewed uniprot protein database.

**Animal experiments**

All animal experiments were approved by the Nanfang Hospital Animal Care and Use Committee and followed the National Guidelines for Animal Experimentation. Four-week-old female BALB/C nude mice received subcutaneous injections of 1 × 10^6^ cells, and tumor nodule volumes were monitored every other day, as previously described.^[1](#_ENREF_1)^ For drug treatment assays, mice were randomly assigned to (without blinding) different treatment groups (three mice per group) when the tumor volume reached approximately 50 mm^3^ and were administered intraperitoneal injections.

**Statistical analyses**

All experiments (with the exception of those involving mice) were performed in at least three independent biological replicates, with technical replicates for each experiment given in the methods. Data were represented as mean ± SD deviation from the independent experiments. Student’s *t-*test (two groups) and one-way Analysis of Variance (ANOVA, more than two groups) were used for comparing groups with normally distributed data; otherwise, the Mann-Whitney U test (two groups) and Kruskal-Wallis test (more than two groups) were performed. The Chi square test was used for the cross test of categorical variables. Survival rates were calculated using the Kaplan–Meier method and examined using the log-rank test. Uni- and multivariate analyses were performed using Cox proportional hazard models, using the “LR forward” stepwise method. All statistical analyses were conducted using SPSS software (version 25.0) and *p* values were two-tailed. Statistical significance was set at *p* < 0.05.
